# Synthesis of Anthraquinone Mono‐ and Diboron Complexes with Near‐Infrared Panchromatic Absorption

**DOI:** 10.1002/chem.202501915

**Published:** 2025-08-19

**Authors:** Yasuhiro Kubota, Ayumi Ogasawara, Shota Mizuno, Toshiyasu Inuzuka, Kazumasa Funabiki

**Affiliations:** ^1^ Department of Chemistry and Biomolecular Science Faculty of Engineering Gifu University 1‐1 Yanagido Gifu 501–1193 Japan; ^2^ Life Science Research Center Gifu University 1‐1 Yanagido Gifu 501–1193 Japan

**Keywords:** anthraquinone, boron complex, near infrared absorption, panchromatic absorption, β‐iminoenolate structure

## Abstract

Anthraquinone monoboron complexes **3** and **4**, possessing a donor–acceptor (D–A) structure, and diboron complexes **7** and **8**, featuring a donor–acceptor–donor (D–A–D) structure, were readily synthesized in two steps from commercially available reagents. These boron complexes adopt a *β*‐iminoenolate structure and their absorption properties are markedly affected by the type of the substituent groups. The unsubstituted monoboron complex **3** (fwhm = 3,950 cm^−1^) and diboron complex **7** (fwhm = 2,510 cm^−1^) exhibited sharp UV–Vis–NIR absorption spectra, which can be attributed to increased molecular rigidity induced by boron coordination. In contrast, the dimethylamino‐substituted monoboron complex **4** (fwhm = 7,580 cm^−1^) and diboron complex **8** (fwhm = 8,770 cm^−1^) showed broad absorption bands due to the enhanced intramolecular charge‐transfer (ICT) character. Dimethylamino‐substituted monoboron complex **4** and diboron complex **8** exhibited panchromatic absorption ranging from 400 nm to 1,000 nm and 400 nm to 1,250 nm, respectively. The panchromatic absorption of the dimethylamino‐substituted boron complexes **4** and **8** is attributed not only to the enhanced ICT character but also to the combination of multiple transitions.

## Graphical abstract

Anthraquinone boron complexes bearing *β*‐iminoenolate ligand(s) exhibit strongly substituent‐dependent absorption properties. Unsubstituted monoboron and diboron complexes show sharp UV–Vis–NIR absorption spectra resulting from increased molecular rigidity induced by boron coordination. In contrast, dimethylamino‐substituted complexes display near‐infrared (NIR) panchromatic absorption, attributed to enhanced intramolecular charge transfer and multiple electronic transitions.

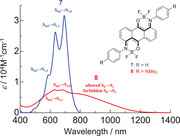

## 1 Introduction

Natural anthraquinones were the most common red natural colorants for textile dyeing from many thousand years ago until the late 19th century.^[^ ^1^ ^]^ Alizarin, one of the anthraquinone derivatives, is the world's first industrially synthesized dyes as a natural dye in 1869 and synthetic anthraquinone derivatives have been widely used as textile dyes^[^ ^2^ ^]^ and food colorants^[^ ^3^ ^]^ even now. Biological properties of anthraquinones such as prevention of fungal and bacterial, antioxidant, anticancer and anti‐inflammatory have also been attracting much attention.^[^ ^4^ ^]^ Application of anthraquinones to colorimetric chemosensors,^[^ ^5^ ^]^ electrochromic materials,^[^ ^6^ ^]^ nonlinear optical (NLO) materials,^[^ ^7^ ^]^ organic semiconductors,^[^ ^8^ ^]^ liquid crystals^[^ ^9^ ^]^ and production of hydrogen peroxide^[^ ^10^ ^]^ have also been reported. The UV–Vis absorption band of anthraquinone dyes depends on the substituents. The absorption maximum (*λ*_max_) of amino derivatives are more bathochromic than the hydroxy derivatives. 1,4,5,8‐Tetraaminoanthraquinone (*λ*_max_: 610 nm in methanol) is most bathochromic followed by 1,4‐diamino (590 nm), and 1,5‐diamino (488 nm) and 1‐amino (475 nm) derivatives.^[^ ^11^ ^]^

Near‐infrared (NIR) absorbing dyes play a crucial role in various applications, including charge‐generation materials, information‐storage materials organic photovoltaics (OPVs), photodetectors (OPDs), and organic field‐effect transistors (OFETs).^[^ ^12^ ^]^ Recently, dyes absorbing near‐infrared‐II light (NIR‐II, 1000–1700 nm) have gained attention in biomedical applications such as photoacoustic imaging (PAI),^[^ ^13^ ^]^ photo dynamic therapy (PDT)^[^ ^14^ ^]^ and photothermal therapy (PTT)^[^ ^15^ ^]^ due to the reduced scattering and minimized autofluorescence in tissues. Cyanines,^[^ ^16^ ^]^ squaraines,^[^ ^17^ ^]^ croconaines,^[^ ^18^ ^]^ xanthenes,^[^ ^19^ ^]^ phthalocyanines,^[^ ^20^ ^]^ rylenes,^[^ ^21^ ^]^ triarylmethanes,^[^ ^22^ ^]^ donor − acceptor dyes,^[^ ^23^ ^]^ and boron complexes^[^ ^24^ ^]^ are known as NIR absorbing dyes. As NIR absorbing anthraquinone dyes, Müellen et al. have reported anthraquinones with two fused rylene dyes (*λ*_max_: 1,000 nm in chloroform)^[^ ^25^ ^]^ and anthraquinones substituted with four aminocarbazoles (*λ*_max_: 777 nm in THF).^[^ ^26^ ^]^ We have also reported anthraquinones fused with two benzothiazines and substituted with two aminoanisoles (*λ*_max_: 800 nm in dichloromethane)^[^ ^27^ ^]^ show NIR absorption. Additionally, panchromatic dyes, known for their efficient sunlight‐harvesting properties, have recently gained attention for applications in solar cells and OPDs.^[^ ^28^ ^]^ In particular, panchromatic dyes that are responsive from UV–Vis to NIR are valuable for applications such as remote control, photometry, day–night monitoring, chemical and biological sensing, and next‐generation wearable electronics.^[^ ^29^ ^]^ However, panchromatic dyes that absorb in the NIR region are still rare.

**Figure 1:**
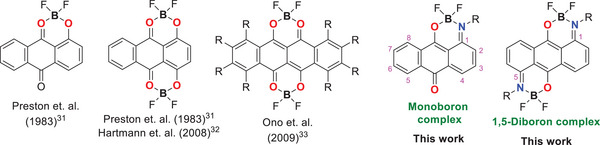
Anthraquinone‐based boron complexes.

On the other hand, recently, boron complexes have attracted much attention due to their excellent optical properties.^[^ ^30^ ^]^ For boron complexes of anthraquinone, although Preston et al.^[^ ^31^ ^]^ and Hartmann et. al^[^ ^32^ ^]^ have reported O^O type six‐membered bidentate boron complexes, the optical properties have not been studied (Figure 1). Ono and coworkers have reported naphthacenequinone‐based boron complexes^[^ ^33^ ^]^ show fluorescence. For N^O type six‐membered bidentate boron complexes, Gorelik and coworkers reported the boron complexes of 1‐(amino)anthraquinone as the intermediates to the synthesis of substituted anthraquinones due to strong electron‐withdrawing ability of the BF_2_ moiety in 1986.^[^ ^34^ ^]^ To the best of our knowledge, the isolation and optical properties of the N^O type six‐membered bidentate boron complexes have not been reported so far. In the course of our study on the boron complexation of dyes,^[^ ^35^ ^]^ we consider that aminoanthraquinones can form BF_2_‐complex dyes and these complex dyes may show unique optical properties. In this study, we report the synthesis of anthraquinone monoboron complexes and 1,5‐diboron complexes having NIR panchromatic absorption property (Figure 1).

### 1.1 Synthesis

**Scheme 1:**
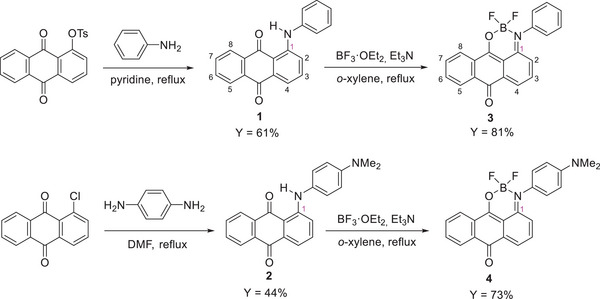
Synthesis of 1‐arylaminoanthraquinone‐based monoboron complexes **3** and **4**.

**Scheme 2:**
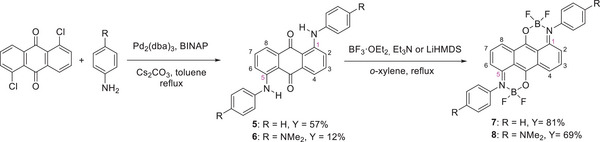
Synthesis of 1,5‐bis(arylamino)anthraquinone‐based diboron complexes **7** and **8**.

1‐Arylaminoanthraquinones **1^[^** ^36^ ^]^ and **2^[^** ^37^ ^]^ and 1,5‐bis(arylamino)anthraquinones **5^[^** ^38^ ^]^ and **6^[^** ^37^ ^]^ are reported known compounds. 1‐Anilinoanthraquinone (**1**) was synthesized by the reaction of 1‐(tosyloxy)anthraquinone with aniline in the presecnce of pyridine (Scheme 1).^[^ ^36^ ^]^ 1‐(4‐Dimethylaminoanilino)anthraquinone (**2**) was synthesized by the nucleophilic reaction between 1‐chloroanthraquinone and dimethyl‐*p*‐phenylenediamine. Müllen and co‐workers reported the synthesis of 1,5‐bis(amino)anthraquinones from 1,5‐dichloroanthraquinone by palladium‐catalyzed Buchwald − Hartwig type amination reaction.^[^ ^26^ ^]^ According to the synthesis conditions, 1,5‐bis(arylamino)anthraquinones **5** and **6** were obtained (Scheme 2). In the Buchwald–Hartwig type amination reaction, the reaction tends to proceed more efficiently with arylamines of higher acidity.^[^ ^39^ ^]^ *p*‐Dimethylaminoaniline (p*K*_a_ = 6.08)^[^ ^40^ ^]^ is less acidic than aniline (p*K*_a_ = 4.60),^[^ ^40^ ^]^ which likely accounts for the lower yield of **6** (Y = 12%) compared to **5** (Y = 57%). The synthesized anthraquinones **1**, **2**, **5** and **6** were allowed to react with boron trifluoride − diethyl ether complex to afford the corresponding boron complexes **3**, **4**, **7** and **8** (Schemes 1 and 2). In the ^13^C NMR spectra of bis(arylamino)anthraquinone derivative **5** in CDCl_3_, a part of the two aryl groups on the N atom were observed as nonequivalent carbon peaks.

**Figure 2:**
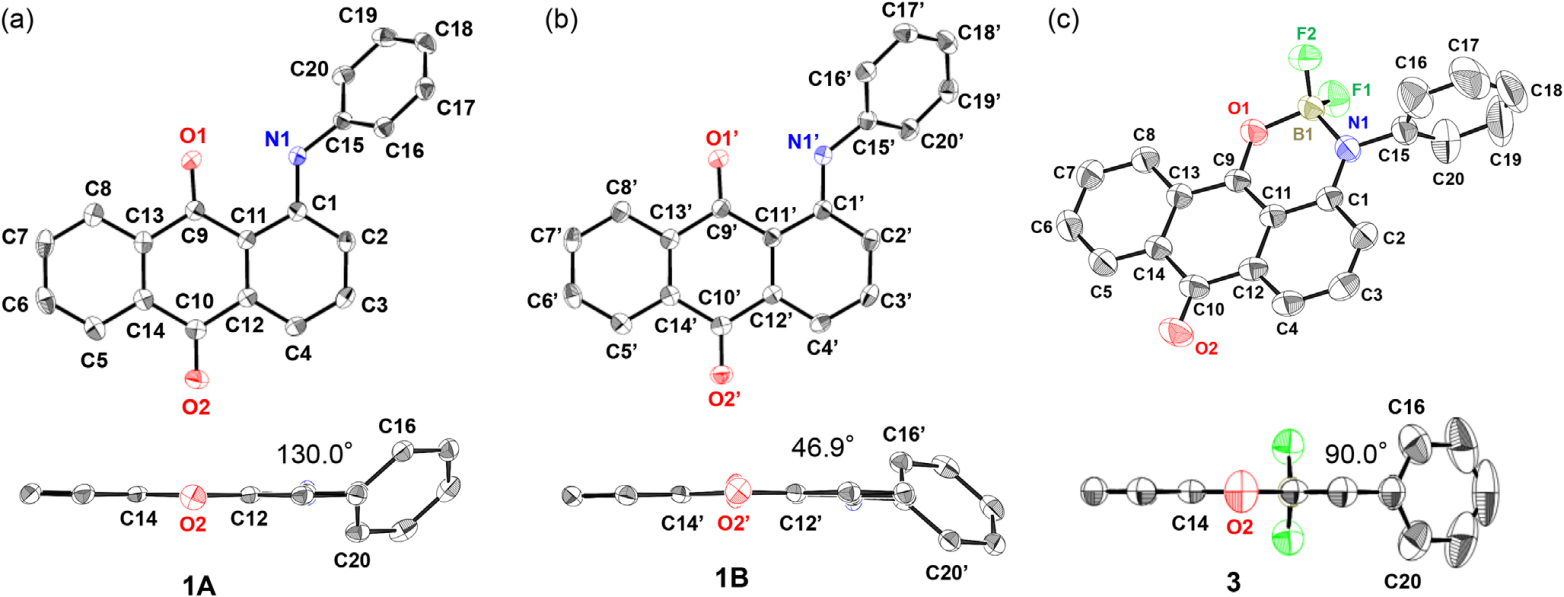
ORTEP drawing of **1** and **3**. a) Top and side views of **1A**. b) Top and side views of **1B**. c) Top and side views of **3**. Hydrogen atoms are omitted for clarity. The dihedral angles between the anthraquinone moiety and the phenyl ring are shown.

**Figure 3:**
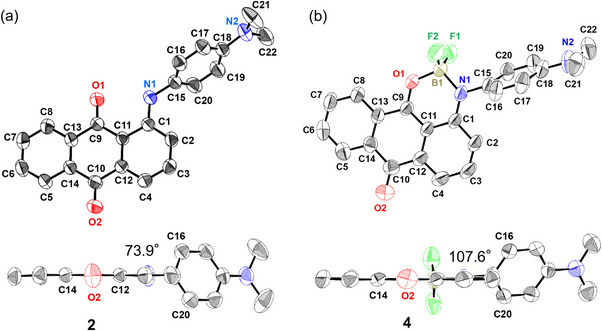
ORTEP drawing of **2** and **4**. a) Top and side views of **2**. b) Top and side views of **4**. Hydrogen atoms are omitted for clarity. The dihedral angles between the anthraquinone moiety and the phenylene moiety are shown.

Single crystals of **1 **− **4** were obtained by slow diffusion of *n*‐hexane into a solution of the corresponding anthraquinones in dichloromethane. The X‐ray crystallographic structures of **1 **− **4** are shown in Figures 2, 3. Experimentally obtained bond lengths of **1 **− **4** in the crystalline state are shown in Figure S1. In the crystal of **1**, two crystallographically independent conformers, **1A** and **1B,** were identified (Figure 2). The biggest difference is the dihedral angle between the anthraquinone moiety and the phenyl group (**1A**: 130.0°, **1B**: 46.9°). The obtained bond lengths of **1A**, **1B,** and **2** were almost the same (Figure S1). Additionally, the bond lengths of anthraquinone monoboron complexes **3** and **4** were almost the same. In contrast, when comparing before and after boron complexation, the C1 − N1 (1.33 Å) and C9 − C11 (1.40 Å) bond lengths of **4** are shorter than the C1 − N1 (1.37 Å) and C9 − C11 (1.47 Å) bond lengths of **2**. In addition, the C9 − O1 (1.30 Å) bond length of **4** is longer than the C9 − O1 (1.23 Å) bond length of **2**. Similar results are obtained when comparing **1** and **3**. These results indicate that the six‐membered N^O bidentate moiety of **3** and **4** adopts the delocalized *β*‐iminoenolate structure rather than the *β*‐ketoiminate structure in the crystalline state (Figure S2). A comparison of the dihedral angles of compounds **3** and **4** shows that **3** adopts a right angle (90°), whereas **4** exhibits a dihedral angle of 107.6°.

### 1.2 UV − Vis − NIR Absorption Properties

#### 1.2.1 Anthraquinone Monoboron Complexes

**Figure 4:**
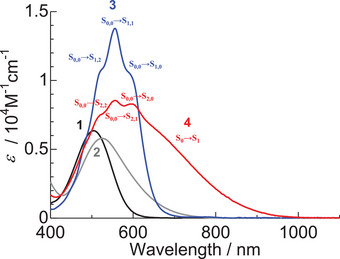
UV − Vis − NIR absorption spectra of 1‐arylaminoanthraquinones **1** and **2** and monoboron complexes **3** and **4** in dichloromethane (3.0 × 10^−5^ M).

**Figure 5:**
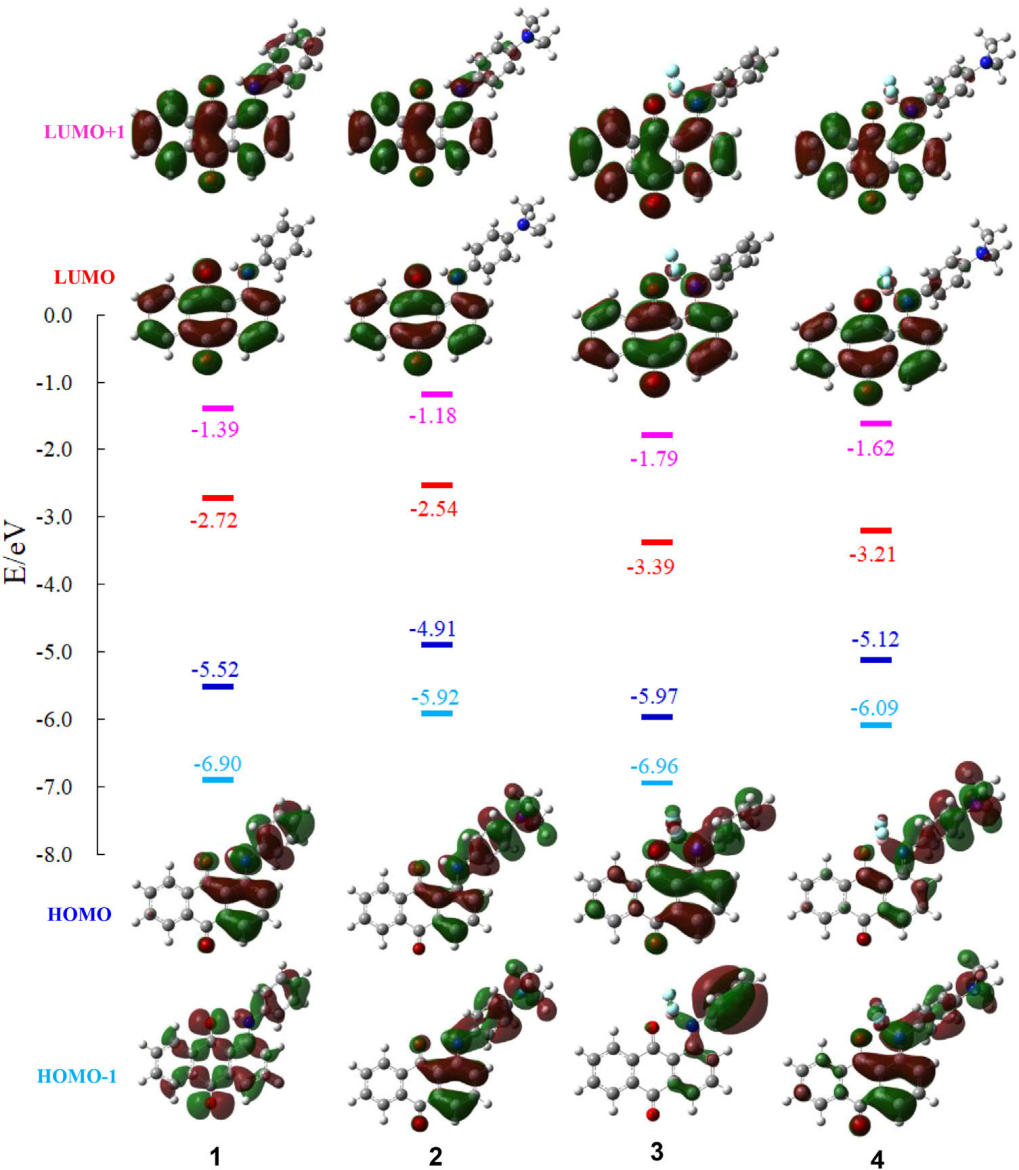
Molecular orbital energy diagrams and isodensity surface plots of **1**–**4**.

The UV − Vis − NIR absorption spectra of **1 **− **4** in dichloromethane are shown in Figure 4. The maximum absorption wavelengths (*λ*_max_) and molar extinction coefficient (*ε*) are listed in Table 1. To obtain a deeper insight into the absorption properties, density functional theory (DFT) calculations were performed.^[^ ^41^ ^]^ Geometry optimization was performed at the B3LYP/6–31G(d,p) level. Electronic transitions were also calculated using the time‐dependent DFT (TDDFT) method^[^ ^42^ ^]^ at the B3LYP/6–31G(d,p) level. The molecular orbital energy diagrams and isodensity surface plots of **1**–**4** are shown in Figure 5. The calculated *λ*_max_ values, principal orbital transitions, and oscillator strength (*f*) values of **1**–**4** are presented in Table 2. The S_0_–S_1_ transition of **1**–**4** was mainly attributed to the transitions from the HOMO to LUMO transition. The calculated molecular orbitals of HOMO and LUMO levels of **1**–**4** were mainly located on the arylamino moiety and the anthraquinone moiety, respectively. Consequently, the S_0_–S_1_ transition of **1**–**4** can be attributed to an intramolecular charge transfer (ICT) transition from the arylamino moiety to the anthraquinone moiety. In other words, compounds **1**–**4** have a donor–acceptor (D–A) structure. DFT calculations indicated that boron complexation primarily resulted in a lowering of the LUMO energy levels, attributable to the electron‐withdrawing ability of the BF_2_ moiety (Figure 5).

**Table 1:** Absorption properties in dichloromethane.

Compd	*λ*_max_ / nm [*ε*]	Fwhm^[^ ^a^ ^]^/cm^−1^
**1**	505 (6300)	4130
**2** ^[^ ^b^ ^]^	526 (5800), 606 (2700)	4980
**3** ^[^ ^b^ ^]^	477 (4800), 516 (10,300), 556 (13,800), 601 (10,000)	3950
**4** ^[^ ^b^ ^]^	479 (5000), 515 (7300), 552 (8500), 604 (8200), 650 (6600)	7580

^[a]^ Full width at half‐maximum height.

^[b]^ The lmax values were extracted using deconvolution of UV − Vis − NIR absorption spectrum.

**Table 2:** Absorption maxima (*λ*_max_), oscillator strengths (*f*) and main orbital transition calculated by TDDFT at the B3LYP/6–31G(d,p) level.

Compd	Transition	*λ*_max_/nm	Main orbital transition	*f*
**1**	S_0_ to S_1_	522	HOMO to LUMO (0.70)	0.14
**2**	S_0_ to S_1_	624	HOMO to LUMO (0.71)	0.11
	S_0_ to S_2_	419	HOMO‐1 to LUMO (0.68)	0.06
**3**	S_0_ to S_1_	544	HOMO to LUMO (0.71)	0.22
**4**	S_0_ to S_1_	780	HOMO to LUMO (0.71)	0.15
	S_0_ to S_2_	487	HOMO‐1 to LUMO (0.70)	0.13

Dimethylamino‐substituted anthraquinone **2** (*λ*_max_ = 526 nm) showed a red‐shifted *λ*_max_ compared to unsubstituted anthraquinone **1** (*λ*_max_ = 505 nm). Additionally, the absorption band of **2** was broader than that of **1**. The full width at half‐maximum height (fwhm) values of **1** and **2** were 4130 and 4980 cm^−1^, respectively. The red‐shifted *λ*_max_ of **2** is attributed to the enhanced electron‐donating ability introduced by the dimethylamino group, which promotes stronger ICT character. TDDFT calculations suggest that the absorption of **1** is attributed solely to the S_0_–S_1_ transition, whereas the absorption of **2** results from a mixture of S_0_–S_1_ and S_0_–S_2_ transitions (Table 2). Therefore, the broadening of the absorption spectrum of **2** can be attributed to both the enhanced ICT character and the overlap of two transitions: S_0_–S_1_ and S_0_–S_2_. Deconvolution of UV − Vis − NIR absorption spectrum of **2** revealed that the S_0_–S_1_ and S_0_–S_2_ transitions are 526 nm and 606 nm, respectively (Table 1 and Figure S3).

Unsubstituted monoboron complex **3** (*λ*_max_ = 556 nm, fwhm = 3950 cm^−1^) showed a red‐shifted *λ*_max_ and sharper absorption band compared to **1**. Unlike compound **1**, monoboron complex **3** exhibited shoulder peaks in its absorption spectrum. The red‐shifted λ_max_ and the sharper absorption band of **3** are attributed to the lowering of the LUMO energy (Figure 5) and increased rigidity, respectively, induced by boron complexation. UV − Vis − NIR absorption measurements of **3** at various concentrations in dichloromethane revealed no changes in the shape of the absorption spectra (Figure S4a), indicating that the observed shoulder peaks are not due to aggregate absorption but rather to vibrational features. In the crystalline state of **3**, the phenyl group on the nitrogen atom and the anthraquinone moiety adopt a perpendicular arrangement, indicating significant steric hindrance between these two moieties (Figure 2). This steric hindrance likely restricts the rotation of the phenyl group in solution. Deconvolution of UV − Vis − NIR absorption spectrum of **3** revealed that the S_0,0_–S_1,0_, S_0,0_–S_1,1_, S_0,0_–S_1,2_, and S_0,0_–S_1,3_ transitions are 601 nm, 556 nm, 516 nm, and 477 nm, respectively (Table 1 and Figure S5).

Dimethylamino‐substituted monoboron complex **4** exhibited a broad absorption range (fwhm = 7580 cm^−1^) with an absorption onset around 1,000 nm. Similar to dimethylamino‐substituted anthraquinone **2**, TDDFT calculations suggest that the absorption of **4** is a combination of S_0_–S_1_ and S_0_–S_2_ transitions (Table 2). Deconvolution of UV − Vis − NIR absorption spectrum of **4** revealed that the S_0_–S_1_ and S_0_–S_2_ transitions are 650 nm and 543 nm, respectively (Figure S6a). Similar to unsubstituted monoboron complex **3**, the absorption spectra of **4** in dichloromethane showed no changes when measured at various concentrations (Figure S4b). Thus, the observed shoulder peaks of **4** around 550 nm are attributed to the vibrational peaks. Deconvolution of UV − Vis − NIR absorption spectrum of **4** in the S_0_–S_2_ transition region revealed that the S_0,0–_S_2,0_, S_0,0_–S_2,1_, S_0,0_–S_2,2_, and S_0,0_–S_2,3_ transitions occur at 604 nm, 552 nm, 515 nm, and 479 nm, respectively (Table 1 and Figure S6b). Compared to compound **2**, monoboron complex **4** exhibited a red‐shifted *λ*_max_ and a broadened absorption spectrum. Boron complexation enhances the electron‐withdrawing properties of the anthraquinone moiety, thereby increasing the ICT character **2**. This leads to the observed red‐shifted and spectral broadening in monoboron complex **4**.

**Figure 6:**
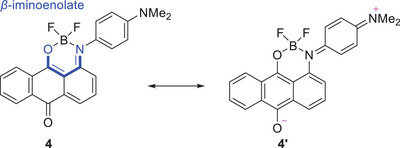
Resonance structure of **4**.

In the dimethylamino‐substituted monoboron complex **4**, the S_0_–S_2_ transition is allowed and assigned to the HOMO–1 to LUMO transition (Table 2). Similar to the S_0_–S_1_ transition, the S_0_–S_2_ transition of **4** corresponds to the ICT transition from the dimethylphenyl moiety to the anthraquinone moiety. A comparison of the HOMO and HOMO–1 orbitals of **4** reveals that the electron density in the HOMO state is significantly more localized at the dimethylaminophenyl moiety than in the HOMO–1 state. Consequently, the S_0_–S_1_ (HOMO to LUMO) transitions of **4** exhibit stronger ICT character compared to the corresponding S_0_–S_2_ (HOMO–1 to LUMO) transitions. Similar to the S_0_–S_1_ (HOMO to LUMO) transition of **3** and the S_0_–S_2_ (HOMO–1 to LUMO) transition of **4** exhibited vibrational peaks (Figure 4). The observed similarity may be attributed to the comparable LUMO orbitals of **3** and **4**, as well as the resemblance between the HOMO orbital of **3** and the HOMO–1 orbital of **4**. Given that **4** adopts a *β*‐iminoenolate structure, the contribution of a resonance structure such as **4′** is plausible (Figure 6). Therefore, the *β*‐iminoenolate structure is likely a key factor contributing to the significant change in absorption properties upon introduction of a dimethylamino group to **3**.

#### 1.2.2 1,5‐Anthraquinone Diboron Complexes

**Figure 7:**
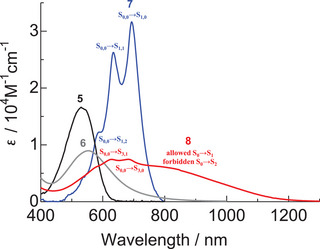
UV − Vis − NIR absorption spectra of 1,5‐bis(arylamino)anthraquinones **5** and **6** and 1,5‐diboron complexes **7** and **8** in dichloromethane (3.0 × 10^−5^ M).

**Figure 8:**
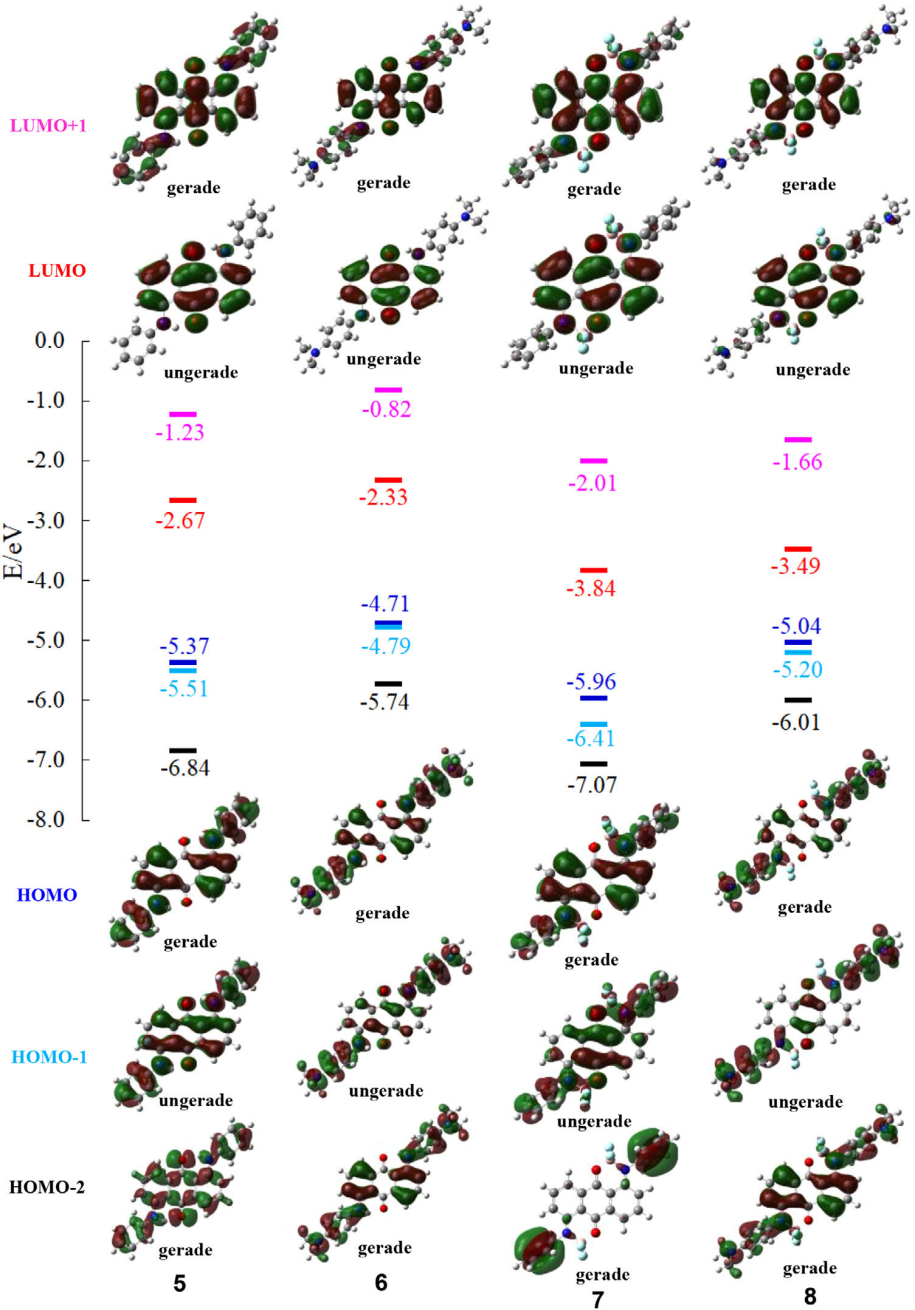
Molecular orbital energy diagrams and isodensity surface plots of **5**–**8**.

Different from **1 **− **4**, compounds **5 **− **8** have high symmetry (*S*_2_ symmetry). Although **1 **− **4** have a D–A structure, **5 **− **8** have a D–A–D structure. The UV − Vis − NIR absorption properties of 1,5‐bis(arylamino)anthraquinones **5** and **6** and 1,5‐diboron complexes **7** and **8** in dichloromethane are shown in Figure 7 and Table 3. Boron‐complexation of **5** and **6** induced a pronounced red‐shift in *λ*_max_. Upon boron‐complexation, the UV–vis–NIR absorption spectra of the unsubstituted derivatives (**5**: fwhm = 3880 cm^−1^, **7**: fwhm = 2510 cm^−1^) became sharper, whereas those of the dimethylamino‐substituted derivatives (**6**: fwhm = 5740 cm^−1^, **8**: fwhm = 8770 cm^−1^) became broader. Interestingly, dimethylamino‐substituted 1,5‐diboron complex **8** showed a wide range of absorption from visible to NIR region, with an absorption onset around 1,250 nm. Similar to **1 **− **4**, geometry optimization and TDDFT calculations of **5 **− **8** were performed using the B3LYP/6–31G(d,p) method in a vacuum, and the results are shown in Figure 8 and Table 4, respectively. The S_0_–S_1_ transition of **5 **− **8** is mainly attributed to the transitions from the HOMO to LUMO transition. The calculated molecular orbitals of HOMO and LUMO levels of **5 **− **8** are mainly located on the two arylamino moieties and the anthraquinone moiety, respectively (Figure 7). Therefore, the S_0_–S_1_ transition of **5 **− **8** corresponds to the ICT transition from the two terminal arylaimino moieties to the central anthraquinone moiety. TDDFT calculations suggest that the absorption of unsubstituted derivatives **5** and **7** is composed solely of the S_0_–S_1_ transition (Table 4). In unsubstituted diboron complex **7**, the experimentally observed absorptions at 694 nm, 634 nm, 583 nm, and 533 nm can be attributed to the S_0,0_–S_1,0_, S_0,0_–S_1,1_, S_0,0_–S_1,2_, and S_0,0_–S_1,3_ transitions, respectively.

**Table 3:** Absorption properties in dichloromethane.

Compd	*λ*_max_/nm [*ε*]	Fwhm^[^ ^a^ ^]^ / cm^−1^
**5**	530 (16,600)	3880
**6** ^[^ ^b^ ^]^	537 (8700), 587 (7800)	5740
**7**	533 (3900), 583 (12,000), 634 (26,300), 694 (31,600)	2510
**8** ^[^ ^b^ ^]^	527 (4000), 575 (6000), 624 (7400), 691 (7400), 832 (5800)	8770

^[a]^ Full width at half‐maximum height.

^[b]^ The lmax values were extracted using deconvolution of UV − Vis − NIR absorption spectrum.

**Table 4:** Absorption maxima (*λ*_max_), oscillator strengths (*f*) and main orbital transition calculated by TDDFT at the B3LYP/6–31G(d,p) level.

Compd	Transition	*λ*_max_/nm	Main orbital transition	*f*
**5**	S_0_ to S_1_	546	HOMO to LUMO (0.70)	0.31
**6**	S_0_ to S_1_	625	HOMO to LUMO (0.71)	0.28
	S_0_ to S_2_	597	HOMO‐1 to LUMO (0.71)	0.00
	S_0_ to S_3_	417	HOMO‐2 to LUMO (0.68)	0.12
**7**	S_0_ to S_1_	637	HOMO to LUMO (0.71)	0.45
**8**	S_0_ to S_1_	935	HOMO to LUMO (0.71)	0.43
	S_0_ to S_2_	863	HOMO‐1 to LUMO (0.71)	0.00
	S_0_ to S_3_	542	HOMO‐2 to LUMO (0.70)	0.19

**Figure 9:**
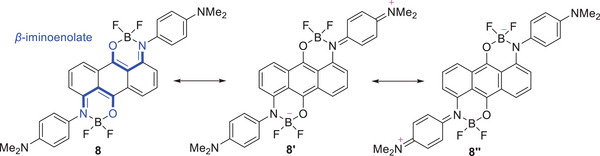
Resonance structure of **8**.

On the other hand, TDDFT calculations suggest that the absorption of dimethylamino‐substituted derivatives **6** and **8** results from the overlap of the S_0_–S_1_ and S_0_–S_3_ transitions (Table 4). The S_0_–S_2_ transition of **6** and **8** was suggested to be forbidden (*f* = 0.00). Since **6** and **8** have a centrosymmetric structure (*S*_2_ symmetry), the Laporte's parity selection rule is applied. According to Laporte's parity selection rule, gerade − gerade and ungerade − ungerade optical transitions are forbidden.^[^ ^43^ ^]^ The S_0_–S_2_ transition of **6** and **8** was mainly attributed to the transitions from the HOMO–1 to LUMO transition. The HOMO − 1 and LUMO orbitals of **6** and **8** are ungerade (Figure 8). Therefore, the forbidden transition between the HOMO − 1 and LUMO can be attributed to the parity forbidden caused by their centrosymmetric structure. The S_0_–S_3_ transition of **6** and **8** was mainly attributed to the transitions from the allowed HOMO − 2 (gerate) to LUMO (ungerade) transition, which is the ICT transition from the two terminal dimethylaminophenyl moieties to the central anthraquinone moiety. Deconvolution of the UV–Vis–NIR absorption spectra of **6** and **8** revealed that the S_0_–S_1_ and S_0_–S_3_ transitions occur at 587 nm and 537 nm, respectively, for **6**, and at 832 nm and 626 nm, respectively, for **8** (Figures S7, S8a). Deconvolution of UV − Vis − NIR absorption spectrum of **8** in the S_0_–S_3_ transition region revealed that the S_0,0_–S_3,0_, S_0,0_–S_3,1_, S_0,0_–S_3,2_, and S_0,0_–S_3,3_ transitions occur at 691 nm, 624 nm, 575 nm, and 527 nm, respectively (Table 3 and Figure S8b). Similar to monoboron complexes **3** and **4**, the experimentally observed S_0_–S_1_ (HOMO to LUMO) transition of **7** and S_0_–S_3_ (HOMO–2 to LUMO) transition of **8** are similar (Figure 7). This similarity may be attributed to the comparable LUMO orbitals of **7** and **8**, as well as the resemblance between the HOMO orbital of **7** and the HOMO–2 orbital of **8**. Since **8** adopts two *β*‐iminoenolate structures, the contributions of resonance structures **8′** and **8′’** are considered plausible (Figure 9). Similar to the monoboron complexes, the *β*‐iminoenolate structures likely play a key role in the significant changes in absorption properties observed upon the introduction of two dimethylamino groups to **7**.

Natural transition orbital (NTO) analysis of boron complexes **3**, **4**, **7,** and **8** was performed at the TDDFT level (B3LYP/6–31G(d,p)) to simplify the description of electronic excitations by representing each excitation as a single pair of occupied and virtual orbitals.^[^ ^44^ ^]^ The NTO analysis provided consistent results with the major electronic configurations obtained from the TDDFT calculations shown in Tables 2 and 4, supporting the nature of the excited states discussed above.

Conventional NIR chromophores suffer from poor photostability.^[^ ^45^ ^]^ To evaluate the durability of the synthesized boron complexes, a photostability test of compounds **4** and **8** was conducted under continuous light irradiation in dichloromethane (Figure S9). These boron complexes were compared with a representative NIR‐absorbing dye, heptamethine cyanine.^[^ ^45^ ^]^ While the cyanine dye was almost completely decomposed after 10 days of irradiation, compounds **4** and **8** showed no detectable decomposition, clearly demonstrating their superior photostability (Figure S10). Boron complexes **3**, **4**, **7**, and **8** exhibited no fluorescence (fluorescence quantum yield = 0.00). Therefore, it was not possible to experimentally determine the radiative and nonradiative rate constants. The high photostability of these nonemissive NIR‐absorbing compounds highlights their potential as durable panchromatic dyes for practical applications.

In summary, we synthesized a series of anthraquinone‐based monoboron and diboron complexes and characterized their structures and optical properties. X‐ray crystallography revealed that boron complexation induces a *β*‐iminoenolate structure in the N^O bidentate moiety. UV–Vis–NIR absorption measurements revealed that boron complexation induces a red‐shift in the absorption maxima. This shift is attributed to the lowering of the LUMO energy level, which results from the strong electron‐withdrawing property of the BF_2_ moiety introduced upon complexation. The UV–Vis–NIR absorption spectra of the unsubstituted 1‐aminoanthraquinone **1** (fwhm = 4130 cm^−1^) and 1,5‐diamino anthraquinone **5** (fwhm = 3880 cm^−1^) became sharper upon boron complexation (**3**: fwhm = 3950 cm^−1^, **7**: fwhm = 2510 cm^−1^), attributed to increased molecular rigidity. In contrast, the spectra of the dimethylamino‐substituted 1‐aminoanthraquinone **2** (fwhm = 4980 cm^−1^) and 1,5‐diamino anthraquinone **6** (fwhm = 5740 cm^−1^) were broadened mainly due to the enhanced ICT character (**4**: fwhm = 7580 cm^−1^, **8**: fwhm = 8770 cm^−1^). The experimentally observed the S_0_–S_1_ transition of **3** (HOMO to LUMO) and the S_0_–S_2_ transition of **4** (HOMO–1 to LUMO) are similar, likely due to the comparable LUMO orbitals and the resemblance between the HOMO of **3** and the HOMO–1 of **4**. Similarly, the S_0_–S_1_ transition of **7** and the S_0_–S_3_ transition of **8** show similar behavior, attributed to the comparable LUMO orbitals and the resemblance between the HOMO of **7** and the HOMO–2 of **8**. Due to its high symmetry (*S*_2_ symmetry), the S_0_–S_2_ transition of **8** becomes forbidden. Dimethylamino‐substituted monoboron complex **4** and diboron complex **8** exhibited panchromatic absorption ranging from 400 nm to 1,000 nm and 400 nm to 1,250 nm, respectively. The panchromatic absorption observed in the dimethylamino‐substituted boron complexes **4** and **8** results from both the enhanced ICT character and the contribution of multiple transitions (**4**: S_0_–S_1_ and S_0_–S_2_, **8**: S_0_–S_1_, forbidden S_0_–S_2_ and S_0_–S_3_). The *β*‐iminoenolate structure of both mono‐ and diboron complexes is likely a crucial factor contributing to the pronounced changes in absorption properties observed upon introduction of dimethylamino groups.

## 2 Experimental Section

### General information

NMR spectra were recorded on ECX400P, JNM‐ECZ600R, and Bruker Biospin AVANCEIII800 spectrometer. Infrared (IR) spectra were determined on a Simazu IR–Affinity spectrometer. UV–Vis–NIR absorption spectra were recorded using a Hitachi U4100 and a Shimadzu UV‐3600i Plus spectrophotometer. Mass spectra were recorded on a JEOL JMS–700 spectrometer. Elemental analyses were performed on a Yanaco MT–6 CHN corder. Melting points were measured on a Yanagimoto MP–S2 micro–melting–point apparatus. Analytical thin‐layer chromatography (TLC) was performed on pre‐coated plates (Merck, silica gel 60 F254). Silica gel (Wakogel C–200) was used for column chromatography.

### X‐ray crystallography

Single‐crystal X‐ray diffraction measurements were carried out with a Rigaku Mercury 375R/M CCD (XtaLAB‐mini) diffractometer with graphite‐monochromated Mo K*α* radiation (*λ* = 0.71 075 Å). The crystal structure was solved using direct method with SHELXT‐2018/2^[^ ^46^ ^]^ and refined by full matrix least‐squares methods with anisotropic thermal parameters for nonhydrogen atoms on *F* ^2^ using SHELXL‐2018/3.^[^ ^47^ ^]^ Hydrogen atoms were placed at calculated positions and refined by applying riding models. The Cambridge Crystallographic Data Center (CCDC) numbers for **1**, **2**, **3,** and **4** are 2 441 010, 2 441 011, 2 441 012, and 2 441 013, respectively.

Crystallographic details for **1**: C_20_H_13_NO_2_, *M* = 299.31 g mol^−1^; *T* = 100(2) K; monoclinic, space group *P*1*n*1; *a* = 14.4579(2), *b* = 3.84340(10), *c* = 26.0061(5) Å, *α* = 90, *β* = 103.794(2), *γ* = 90°; *V* = 1403.42(5) Å^3^; *Z* = 4; 3.216 < *θ* < 77.000; *ρ*_calcd_ = 1.417 g cm^−3^; *μ* = 0.737 mm^−1^; *R*_int_ = 0.0449; *R*_1_ = 0.0369, w*R*_2_ = 0.0968 for 4556 reflections with *I* > 2*σ*(I) and 415 parameters; *S* = 1.048; CCDC 2 441 010.

Crystallographic details for **2**: C_22_H_18_N_2_O_2_, *M* = 342.38 g mol^−1^; *T* = 293(2) K; monoclinic, space group *P*2_1_/*a*; *a* = 8.664(2), *b* = 16.429(4), *c* = 12.307(4) Å, *α* = 90, *β* = 98.985(15), *γ* = 90°; *V* = 1730.3(8) Å^3^; *Z* = 4; 2.961 < *θ* < 27.575; *ρ*_calcd_ = 1.314 g cm^−3^; *μ* = 0.085 mm^−1^; *R*_int_ = 0.0385; *R*_1_ = 0.0640, w*R*_2_ = 0.2050 for 3907 reflections with *I* > 2*σ*(I) and 239 parameters; *S* = 1.069; CCDC 2 441 011.

Crystallographic details for **3**: C_20_H_12_BF_2_NO_2_, *M* = 347.12 g mol^−1^; *T* = 293(2) K; orthorhombic, space group *Pnma*; *a* = 14.71(3), *b* = 7.017(14), *c* = 15.27(3) Å, *α* = 90, *β* = 90, *γ* = 90°; *V* = 1576(5) Å^3^; *Z* = 4; 2.668 < *θ* < 27.428; *ρ*_calcd_ = 1.463 cm^−3^; *μ* = 0.110 mm^−1^; *R*_int_ = 0.0254; *R*_1_ = 0.0496, w*R*_2_ = 0.1425 for 1875 reflections with *I* > 2*σ*(I) and 148 parameters; *S* = 1.067; CCDC 2 441 012.

Crystallographic details for **4**: C_22_H_17_BF_2_N_2_O_2_, *M* = 390.18 g mol^−1^; *T* = 293(2) K; triclinic, space group *P*‐1; *a* = 8.5822(18), *b* = 9.623(2), *c* = 12.917(4) Å, *α* = 101.46(7), *β* = 105.01(7), *γ* = 110.84(9)°; *V* = 911.6(7) Å^3^; *Z* = 2; 2.646 < *θ* < 27.661; *ρ*_calcd_ = 1.421 cm^−3^; *μ* = 0.105 mm^−1^; *R*_int_ = 0.0275; *R*_1_ = 0.0527, w*R*_2_ = 0.1652 for 4127 reflections with *I* > 2*σ*(I) and 263 parameters; *S* = 0.948; CCDC 2 441 013.

### Synthesis of 1‐anilinoanthraquinone (1)^[^ ^36^ ^]^

A solution of 1‐(tosy1oxy)anthraquinone (2.50 g, 6.61 mmol) and aniline (30 mL, 0.33 mol) in pyridine (30 mL) was refluxed for 4 days. Water was added to the reaction mixture, extracted with DCM, and washed with 2 M HCl, 2 M NaOH, and water. The organic layer was dried over Na_2_SO_4_ and evaporated under reduced pressure. The residue was purified by column chromatography on silica gel (DCM: Hexane = 2: 1) to give **1** (1.21 g, 61%) as a red solid. The single crystals of **1** were obtained by slow diffusion of hexane into a solution in DCM. ^1^H NMR (400 MHz, CDCl_3_) *δ* 7.22 (t, *J* = 7.4 Hz, 1H), 7.33 (d, *J* = 7.4 Hz, 2H), 7.43 (dd, *J* = 7.4, 7.4 Hz, 2H), 7.51 (d, *J* = 8.2 Hz, 1H), 7.52 (d, *J* = 8.7 Hz, 1H), 7.73 (dd, *J* = 8.7, 8.2 Hz, 1H), 7.75 (ddd, *J* = 7.6, 7.6, 1.3 Hz, 1H), 7.80 (ddd, *J* = 7.6, 7.6, 1.3 Hz, 1H), 8.28 (dd, *J* = 7.6, 1.3 Hz, 1H), 8.33 (dd, *J *= 7.6, 1.3 Hz, 1H), 11.37 (s, 1H); ^13^C NMR (100 MHz, CDCl_3_) *δ* 114.12, 117.79, 120.02, 124.17, 124.31, 125.08, 125.21, 126.89, 129.57, 133.06, 133.30, 134.06, 134.66, 134.84, 139.36, 149.31, 183.49, 185.46; IR (KBr) 3229, 3067, 1663, 1628, 1589, 1504, 1362, 1304, 1269, 1231, 1165, 1072, 1015, 706 cm^−1^.

### Synthesis of 1‐(4‐dimethylaminoanilino)anthraquinone (2)^[^ ^37^ ^]^

A solution of 1‐chloroanthraquinone (3.00 g, 12.4 mmol) and *N*,*N*‐dimethyl‐*p*‐phenylenediamine (11.8 g, 86.6 mmol) in DMF (100 mL) was refluxed for 1 day. Water was added to the reaction mixture, and the resulting precipitate was filtered and washed with methanol. Column chromatography of the residue on silica gel (DCM) gave **2** (1.88 g, Y = 44%) as a purple solid. The single crystals of **2** were obtained by slow diffusion of hexane into a solution in DCM. ^1^H NMR (400 MHz, CDCl_3_) *δ* 2.99 (s, 6H), 6.78 (d, *J* = 9.2 Hz, 2H), 7.18 (d, *J* = 9.2 Hz, 2H), 7.29 (dd, *J* = 8.7, 1.4 Hz, 1H), 7.43 (dd, *J* = 8.7, 7.4 Hz, 1H), 7.65 (dd, *J* = 7.4, 1.4 Hz, 1H), 7.73 (ddd, *J* = 7.8, 7.8, 1.4 Hz, 1H), 7.79 (ddd, *J* = 7.8, 7.8, 1.4 Hz, 1H), 8.27 (dd, *J* = 7.8, 1.4 Hz, 1H), 8.33 (dd, *J* = 7.8, 1.4 Hz, 1H), 11.18 (s, 1H); ^13^C NMR (100 MHz, CDCl_3_) *δ* 40.77, 113.17, 113.27, 116.90, 120.09, 126.72, 126.77 (2C), 128.05, 133.02, 133.11, 133.97, 134.49, 134.79, 135.04, 148.85, 151.16, 183.73, 185.00; IR (KBr) 3248, 3079, 1663, 1628, 1589, 1520, 1497, 1304, 1269, 1234, 1072, 737, 710 cm^−1^.

### Synthesis of 3

Triethylamine (0.46 mL, 3.3 mmol) and boron trifluoride diethyl ether complex (1.7 mL, 13 mmol) were added to a solution of **1** (101 mg, 0.337 mmol) in *o‐*xylene (30 mL). After refluxing for 3 hours, water was added, extracted with DCM, washed with water, dried over Na_2_SO_4_ and evaporated under reduced pressure. Column chromatography of the residue on silica gel (DCM) gave **3** (95 mg, Y = 81%) as a purple solid. The single crystals of **3** were obtained by slow diffusion of hexane into a solution in DCM. mp 254 − 256 °C; ^1^H NMR (600 MHz, CDCl_3_) *δ* 6.95 (d, *J* = 9.6 Hz, 1H), 7.41 (d, *J* = 7.6 Hz, 2H), 7.47 (t, *J* = 7.6 Hz, 1H), 7.53 − 7.57 (m, 3H), 7.72 (d, *J* = 6.2 Hz, 1H), 7.89 − 7.91 (m, 2H), 8.37 − 8.39 (m, 1H), 8.54 − 8.56 (m, 1H); ^13^C NMR (100 MHz, CDCl_3_) *δ* 109.28, 121.77, 123.52, 127.48, 127.92, 128.10, 128.16, 129.79, 131.08, 131,97, 133.83, 134.59, 135.31, 139.43, 139.84, 154.91, 173.37, 181.07; ^19^F NMR (564 MHz, CDCl_3_) *δ* ‐125.50 (q, *J* = 19.7 Hz); ^11^B NMR (193 MHz, CDCl_3_) *δ* 0.54 (t, *J* = 19.7 Hz); IR (KBr) 3093, 1670, 1616, 1589, 1535, 1477, 1454, 1288, 1107, 1065, 1018, 702 cm^−1^; EIMS (70 eV) *m/z* (rell intensity) 347 (M^+^, 100); Anal. Found: C, 69.42; H, 3.48; N, 4.07%. Calcd for C_20_H_12_BF_2_NO_2_: C, 69.20; H, 3.48; N, 4.04%.

### Synthesis of 4

Triethylamine (0.4 mL, 2.9 mmol) and boron trifluoride diethyl ether complex (1.8 mL, 14 mmol) were added to a solution of **2** (103 mg, 0.301 mmol) in *o‐*xylene (120 mL). After refluxing for 3 hours, water was added, extracted with DCM, washed with water, dried over Na_2_SO_4_ and evaporated under reduced pressure. Column chromatography of the residue on silica gel (DCM) gave **4** (86 mg, Y = 73%) as a purple solid. The single crystals of **4** were obtained by slow diffusion of hexane into a solution in DCM. mp: 264 − 265 °C; ^1^H NMR (400 MHz, CDCl_3_) *δ* 3.02 (s, 6H), 6.80 (d, *J* = 9.2 Hz, 2H), 7.05 (dd, *J* = 9.6, 0.9 Hz, 1H), 7.18 (d, *J* = 9.2 Hz, 2H), 7.47 (dd, *J* = 9.2, 6.4 Hz 1H), 7.67 (d, *J* = 6.4 Hz, 1H), 7.80 − 7.87 (m, 2H), 8.33 (dd, *J* = 5.0, 2.8 Hz, 1H), 8.50 (dd, *J* = 6.9, 2.3 Hz, 1H); ^13^C NMR (100 MHz, CDCl_3_) *δ* 40.54, 109.28, 112.89, 121.95, 124.25, 127.68, 127.70, 128.06, 128.21, 131.31, 131.86, 133.70, 134.47, 134.81, 139.11, 150.04, 155.17, 171.76, 181.22; ^19^F NMR (564 MHz, CDCl_3_) *δ* ‐126.33 (q, *J* = 18.1 Hz); ^11^B NMR (193 MHz, CDCl_3_) *δ* 0.58 (t, *J* = 18.1 Hz); IR (KBr) 3098, 1667, 1616, 1589, 1524, 1477, 1450, 1292, 1184, 1065, 702 cm^−1^; EIMS (70 eV) *m/z* (rel intensity) 390 (M^+^), 372 (31), 342 (17); Anal. Found: C, 67.54; H, 4.47; N, 7.28%. Calcd for C_22_H_17_BF_2_N_2_O_2_: C, 67.72; H, 4.39; N, 7.18%.

### Synthesis of 1,5‐bis(anilino)anthraquinone (5)^[^ ^38^ ^]^

A toluene (10 mL) solution of 1,5‐dichloroanthraquinone (504 mg, 1.82 mmol), BINAP (88.8 mg, 0.143 mmol), Cs_2_CO_3_ (2.31 g, 7.09 mmol), aniline (372 mg, 3.99 mmol), and Pd_2_(dba)_3_ (44.5 mg, 0.0486 mmol) was refluxed for 5 days. Water was added, extracted with DCM, washed with water, dried over Na_2_SO_4_, and evaporated under reduced pressure. Column chromatography of the residue on silica gel (DCM: hexane = 2: 1) gave **5** (402 mg, Y = 57%) as a purple solid. ^1^H NMR (400 MHz, CDCl_3_) *δ* 7.21 (t, *J* = 7.3 Hz, 2H), 7.33 (d, *J* = 7.3 Hz, 4H), 7.42 (dd, *J* = 7.3, 7.3 Hz, 4H), 7.48 − 7.50 (m, 4H), 7.72 (dd, *J* = 5.7, 3.0 Hz, 2H), 11.4 (s, 2H); ^13^C NMR (100 MHz, CDCl_3_) *δ* 114.08, 117.21, 118.89, 124.00, 124.07, 124.88, 124.99, 129.52, 129.59, 134.89, 134.94, 136.09, 139.54, 148.96, 185.53; IR (KBr) 3232, 3059, 1624, 1589, 1504, 1362, 1307, 1261, 1180, 872, 756, 689 cm^−1^.

### Synthesis of 1,5‐bis(4‐dimethylaminoanilino)anthraquinone (6)^[^ ^37^ ^]^

A toluene (250 mL) solution of 1,5‐dichloroanthraquinone (3.00 g, 10.8 mmol), BINAP (533 mg, 0.856 mmol), Cs_2_CO_3_ (13.9 g, 42.7 mmol), *N*,*N*‐dimethyl‐*p*‐phenylenediamine (3.24 g, 23.8 mmol), and Pd_2_(dba)_3_ (268 mg, 0.293 mmol) was refluxed. Water was added to the reaction mixture and the resulting precipitate was filtered and washed with water and methanol. Water was added, extracted with DCM, washed with water, dried over Na_2_SO_4_ and evaporated under reduced pressure. Column chromatography of the residue on silica gel (DCM: AcOEt: Et_3_N = 200: 5: 2) gave **6** (634 mg, Y = 12%) as a purple solid. ^1^H NMR (400 MHz, CDCl_3_) *δ* 2.99 (s, 12H), 6.78 (d, *J* = 9.1 Hz, 4H), 7.19 (d, *J* = 9.1 Hz, 4H), 7.23 (d, *J* = 8.0 Hz, 2H), 7.42 (dd, *J* = 8.0, 8.0 Hz, 2H), 7.64 (d, *J* = 8.0 Hz, 2H), 11.2 (s, 2H); ^1^H NMR (400 MHz, TFA‐*d*) *δ*  3.46 (s, 12H), 7.61 − 7.72 (m, 12H), 7.83 (d, *J* = 6.8 Hz, 2H); ^13^C NMR (100 MHz, TFA‐*d*) *δ* 47.57, 114.81, 120.57, 121.12, 121.37, 125.25, 135.85, 136.88, 137.99, 142.43, 148.58, 187.62; IR (KBr) 3244, 3078, 1620, 1593, 1566, 1520, 1493, 1450, 1362, 1308, 1258, 1184, 1165, 810, 771, 714 cm^−1^.

### Synthesis of 7

Triethylamine (0.21 mL, 1.51 mmol) and boron trifluoride diethyl ether complex (0.96 mL, 7.62 mmol) were added to a solution of **5** (58.5 mg, 0.150 mmol) in *o‐*xylene (60 mL). After refluxing for 5 hours, water was added, extracted with DCM, washed with water, dried over Na_2_SO_4_ and evaporated under reduced pressure. Column chromatography of the residue on silica gel (DCM: hexane = 4: 1) gave **7** (58.8 mg, Y = 81%) as a blue solid. mp > 300 °C; ^1^H NMR (400 MHz, CDCl_3_) *δ* 6.96 (d, *J* = 9.6 Hz, 2H), 7.38 (d, *J* = 7.3 Hz, 4H), 7.46 (t, *J* = 7.3 Hz, 2H), 7.52 − 7.56 (m, 6H), 7.99 (d, *J* = 6.9 Hz, 2H); ^13^C NMR (200 MHz, CDCl_3_) *δ* 108.72, 124.12, 124.82, 127.26, 128.44, 129.85, 130.03, 139.09, 139.56, 155.63, 168.75; ^19^F NMR (564 MHz, CDCl_3_) *δ* ‐125.80 (q, *J* = 18.1 Hz); ^11^B NMR (193 MHz, CDCl_3_) *δ* 0.27 (t, *J* = 18.1 Hz); IR (KBr) 3090, 1605, 1562, 1528, 1524, 1466, 1312, 1285, 1196, 1130, 1069, 1026, 814, 764, 702 cm^−1^; EIMS (70 eV) *m/z* (rell intensity) 486 (M^+^, 100); Anal. Found: C, 64.32; H, 3.47; N, 5.78%. Calcd for C_26_H_16_B_2_F_4_N_2_O_2_: C, 64.25; H, 3.32; N, 5.76%.

### Synthesis of 8

Lithium bis(trimethylsilyl)amide (57.2 mg, 0.342 mmol) and boron trifluoride diethyl ether complex (0.32 mL, 2.54 mmol) were added to a solution of **6** (30 mg, 0.0629 mmol) in *o‐*xylene (200 mL). After refluxing for 8 hours, the resulting insoluble material was collected by filtration and washed with water and hexane to give **8** (25 mg, Y = 69%) as a blue solid. mp > 300 °C, ^1^H NMR (400 MHz, CDCl_3_) *δ* 3.04 (s, 12H), 6.81 (d, *J* = 8.7 Hz, 4H), 7.10 (d, *J* = 9.2 Hz, 2H), 7.25 (d, *J* = 8.7 Hz, 4H), 7.48 (dd, *J* = 9.2, 7.3 Hz, 2H), 7.95 (d, *J* = 7.3 Hz, 2H); ^13^C NMR (200 MHz, CDCl_3_) *δ* 40.5, 108.9, 112.8, 123.8, 124.9, 127.6, 128.2, 130.1, 138.6, 149.8, 155.5, 166.8; ^19^F NMR (564 MHz, CDCl_3_) *δ* ‐127.50 (q, *J* = 18.6 Hz); ^11^B NMR (193 MHz, CDCl_3_) *δ* 0.48 (t, *J* = 18.6 Hz); IR (KBr) 3082, 1609, 1589, 1520, 1462, 1362, 1319, 1258, 1169, 1123, 1083, 1034 cm^−1^; EIMS (70 eV) *m/z* (rell intensity) 572 (M^+^, 100); Anal. Found: C, 63.12; H, 4.65; N, 9.52%. Calcd for C_30_H_26_B_2_F_4_N_4_O_2_: C, 62.98; H, 4.58; N, 9.79%.

## Conflict of Interest

The authors declare no conflict of interest.

## Supporting information



Supporting Information

Supporting Information

## Data Availability

The data that support the findings of this study are available in the supplementary material of this article.

## References

[1] a) M. Shahid , J. Wertz , I. Degano , M. Aceto , M. I. Khan , A. Quye , Anal. Chim. Acta 2019, 1083, 58; 31493810 10.1016/j.aca.2019.07.009

[2] a) S. Benkhaya , S. M'rabet , A. E. Harfi , Inorg. Chem. Commun. 2020, 115, 107891;

[3] L. Dufossé , Food Res. Int. 2014, 65, 132.

[4] a) J. S. Al‐Otaibi , T. M. EL Gogary , J. Mol. Struct. 2017, 1130, 799;

[5] a) P. Kumar , A. Ghosh , D. A. Jose , Analyst 2019, 144, 594; 30427334 10.1039/c8an01042k

[6] a) Y. Li , M. Zhou , Z. Yang , Y. Li , J. Mater. Sci. 2018, 53, 15600;

[7] P. O. Gupta , N. Sekar , ChemistrySelect 2024, 9, e202400736.

[8] a) M. Zhao , X. Yang , G. C. Tsui , Q. Miao , J. Org. Chem. 2020, 85, 44; 31309837 10.1021/acs.joc.9b01263

[9] a) E. I. L. Jull , M. Wahle , P. J. M. Wyatt , C. Ellis , S. J. Cowling , J. W. Goodby , K. Usami , H. F. Gleeson , Opt. Express 2019, 27, 26799; 31674554 10.1364/OE.27.026799

[10] a) Q. Zhu , Z. Pan , S. Hu , J.‐H. Kim , ACS Appl. Energy Mater. 2019, 2, 7972;

[11] A. N. Dias , J. Photochem. Photobiol. A 1990, 53, 141.

[12] a) D. Meng , R. Zheng , Y. Zhao , E. Zhang , L. Dou , Y. Yang , Adv. Mater. 2022, 34, 2107330; 10.1002/adma.202107330 34710251

[13] H. J. Knox , J. Chan , Acc. Chem. Res. 2018, 51, 2897. 30379532 10.1021/acs.accounts.8b00351 PMC7303888

[14] P. C. A. Swamy , G. Sivaraman , R. N. Priyanka , S. O. Raja , K. Ponnuvel , J. Shanmugpriya , A. Gulyani , Coord. Chem. Rev. 2020, 411, 213233.

[15] S. Lee , S. Min , G. Kim , S. Lee , Coord. Chem. Rev. 2024, 506, 215719.

[16] a) B. Mourot , D. Jacquemin , O. Siri , S. Pascal , Chem. Rec. 2024, 24, e202400183; 39529436 10.1002/tcr.202400183

[17] a) A. Mei , X. He , D. Lei , L. Wang , W. Wang , J. Shao , Q. Shen , F. Jiang , X. Dong , Coord. Chem. Rev. 2025, 527, 216419;

[18] a) T. Maeda , T. Oka , D. Sakamaki , H. Fujiwara , N. Suzuki , S. Yagi , T. Konishi , K. Kamada , Chem. Sci. 2023, 14, 1978; 36845939 10.1039/d2sc06612b PMC9944335

[19] R. Wei , Y. Dong , X. Wang , J. Li , Z. Lei , Z. Hu , J. Chen , H. Sun , H. Chen , X. Luo , X. Qian , Y. Yang , J. Am. Chem. Soc. 2023, 145, 12013. 37216464 10.1021/jacs.3c00594

[20] a) A. Muranaka , M. Uchiyama , Bull. Chem. Soc. Jpn. 2021, 94, 872;

[21] a) L. Jiao , Y. Zou , W. Fan , Y. Han , Q. Zhou , J. Shao , J. Wu , J. Am. Chem. Soc. 2025, 147, 9415; 40053379 10.1021/jacs.4c16524

[22] X. Zhang , M. Liu , Y. Hu , X. Wang , R. Wei , C. Yao , C. Shi , Y. Qiu , T. Yang , X. Luo , J. Chen , W. Sun , H. Chen , X. Qian , Y. Yang , Adv. Mater. 2025, 37, 2411515. 10.1002/adma.202411515 39520340

[23] a) Y. Zeng , J. Qu , G. Wu , Y. Zhao , J. Hao , Y. Dong , Z. Li , J. Shi , J. S. Francisco , X. Zheng , J. Am. Chem. Soc. 2024, 146, 9888; 38546165 10.1021/jacs.3c14805

[24] a) S. Yokoyama , S. Utsunomiya , T. Seo , A. Saeki , Y. Ie , Adv. Sci. 2024, 11, 2405656; 10.1002/advs.202405656 PMC11336916 38873872

[25] C. Kohl , S. Becker , K. Müllen , Chem. Commun. 2002, 2778. 10.1039/b208855j 12478742

[26] A. K. Mishra , J. Jacob , K. Müllen , Dyes Pigm. 2007, 75, 1.

[27] M. Matsui , S. Taniguchi , M. Suzuki , M. Wang , K. Funabiki , H. Shiozaki , Dyes Pigm. 2005, 65, 211.

[28] a) S. Hiroto , M. Chujo , Chem. Asian J. 2025, 20, e202400830; 39215744 10.1002/asia.202400830

[29] a) C. Fuentes‐Hernandez , W. F. Chou , T. M. Khan , L. Diniz , B. Kippelen , Science 2020, 370, 698; 33154137 10.1126/science.aba2624

[30] a) S. Sultan , L. Crovetto , R. Rios , Chem. Commun. 2025, 61, 1989; 10.1039/d4cc05809g 39752291

[31] a) P. N. Preston , T. Winwick , J. Chem. Soc. Perkin Trans. 1 1983, 1983, 1439;

[32] Y. Zhou , Y. Zheng , O. Zeika , H. Hartmann , K. Leo , Mater. Chem. Phys. 2008, 112, 577.

[33] a) K. Ono , J. Hashizume , H. Yamaguchi , M. Tomura , J. Nishida , Y. Yamashita , Org. Lett. 2009, 11, 4326; 19711907 10.1021/ol901633q

[34] M. V. Gorelik , N. N. Shapet'ko , L. V. Arinich , A. I. Tsurkan , M. L. Kukushkina , Zh. Org. Khim. 1986, 22, 611.

[35] a) Y. Kubota , M. Tsukamoto , K. Ohnishi , J. Jin , K. Funabiki , M. Matsui , Org. Chem. Front. 2017, 4, 1522;

[36] A. G. Zielske , J. Org. Chem. 1987, 52, 1305.

[37] M. Ichikawa , M. Okazaki , Kogyo Kagaku Zasshi 1964, 67, 142.

[38] L. He , H. S. Freeman , L. Lu , S. Zhang , Dyes Pigm. 2011, 91, 389.

[39] S. D. McCann , E. C. Reichert , P. L. Arrechea , S. L. Buchwald , J. Am. Chem. Soc. 2020, 142, 15027. 32786769 10.1021/jacs.0c06139 PMC8057821

[40] M. R. Biscoe , T. E. Barder S. L. Buchwald , Angew. Chem. Int. Ed. 2007, 46, 7232. 10.1002/anie.200702122 17680583

[41] M. J. Frisch , G. W. Trucks , H. B. Schlegel , G. E. Scuseria , M. A. Robb , J. R. Cheeseman , G. Scalmani , V. Barone , B. Mennucci , G. A. Petersson , H. Nakatsuji , M. Caricato , X. Li , H. P. Hratchian , A. F. Izmaylov , J. Bloino , G. Zheng , J. L. Sonnenberg , M. Hada , M. Ehara , K. Toyota , R. Fukuda , J. Hasegawa , M. Ishida , T. Nakajima , Y. Honda , O. Kitao , H. Nakai , T. Vreven , J. A. Montgomery Jr. , J. E. Peralta , F. Ogliaro , M. Bearpark , J. J. Heyd , E. Brothers , K. N. Kudin , V. N. Staroverov , R. Kobayashi , J. Normand , K. Raghavachari , A. Rendell , J. C. Burant , S. S. Iyengar , J. Tomasi , M. Cossi , N. Rega , J. M. Millam , M. Klene , J. E. Knox , J. B. Cross , V. Bakken , C. Adamo , J. Jaramillo , R. Gomperts , R. E. Stratmann , O. Yazyev , A. J. Austin , R. Cammi , C. Pomelli , J. W. Ochterski , R. L. Martin , K. Morokuma , V. G. Zakrzewski , G. A. Voth , P. Salvador , J. J. Dannenberg , S. Dapprich , A. D. Daniels , O. Farkas , J. B. Foresman , J. V. Ortiz , J. Cioslowski , D. J. Fox , Gaussian 09, Revision A. 02, Gaussian, Inc., Wallingford, CT, 2009.

[42] a) R. E. Stratmann , G. E. Scuseria , M. J. Frisch , J. Chem. Phys. 1998, 109, 8218;

[43] a) X. Wang , Y. Zhou , T. Lei , N. Hu , E.‐Q. Chen , J. Pei , Chem. Mater. 2010, 22, 3735;

[44] a) A. K. Mandal , J. R. Diers , D. M. Niedzwiedzki , G. Hu , R. Liu , E. J. Alexy , J. S. Lindsey , D. F. Bocian , D. Holten , J. Am. Chem. Soc. 2017, 139, 17547; 29160700 10.1021/jacs.7b09548

[45] Y. Yamada , A. Okamoto , S. Mizuno , T. Udagawa , T. Agou , Y. Kubota , T. Inuzuka , K. Funabiki , ChemPhotoChem 2025, e202500006. 10.1002/cptc.202500006

[46] G. M. Sheldrick , Acta Crystallogr. Sect. A: Found. Adv. 2015, 71, 3. 25537383 10.1107/S2053273314026370 PMC4283466

[47] G. M. Sheldrick , Acta Crystallogr. Sect. C: Struct. Chem. 2015, 71, 3. 25567568 10.1107/S2053229614024218 PMC4294323

