# Supplementary material for: Synthesis of Anthraquinone Mono‐ and Diboron Complexes with Near‐Infrared Panchromatic Absorption
Source: Chemistry. 2025 Aug 19;31(53):e01915. doi: 10.1002/chem.202501915 (PMC12451423; doi:10.1002/chem.202501915)
Supplement: Supplementary file 1 — Supporting Information [file CHEM-31-e01915-s001.pdf]

Supporting information

Synthesis of Anthraquinone Mono- and Diboron Complexes with Near-Infrared Panchromatic Absorption

Yasuhiro Kubota,[a,]* Ayumi Ogasawara,[a] Shota Mizuno,[a] Toshiyasu Inuzuka,[b] Kazumasa Funabiki[a]

[a]*Department of Chemistry and Biomolecular Science, Faculty of Engineering, Gifu University, 1-1 Yanagido, Gifu, 501-1193, Japan*

[b]*Life Science Research Center, Gifu University, 1-1 Yanagido, Gifu 501-1193, Japan*

Contents

1. Experimentally obtained bond lengths of **1–4** (**Figure S1**)
2. Resonance structures between the *β*-iminoenolate and *β*-ketoiminate forms (**Figure S2**).
3. Deconvolution of UV−Vis−NIR absorption spectrum of **2** (**Figure S3**)
4. The effect of concentration on the UV−Vis−NIR absorption spectra of **3** and **4** in dichloromethane (**Figure S4**)
5. Deconvolution of UV−Vis−NIR absorption spectrum of **3**, **4**, **6** and **8** (**Figure S5–S8**)
6. Photostabilities of **4**, **8** and heptamethine cyanine (**Figure S9–S10**)
7. NMR spectra of **1–8** (**Figure S11–Figure S31**)

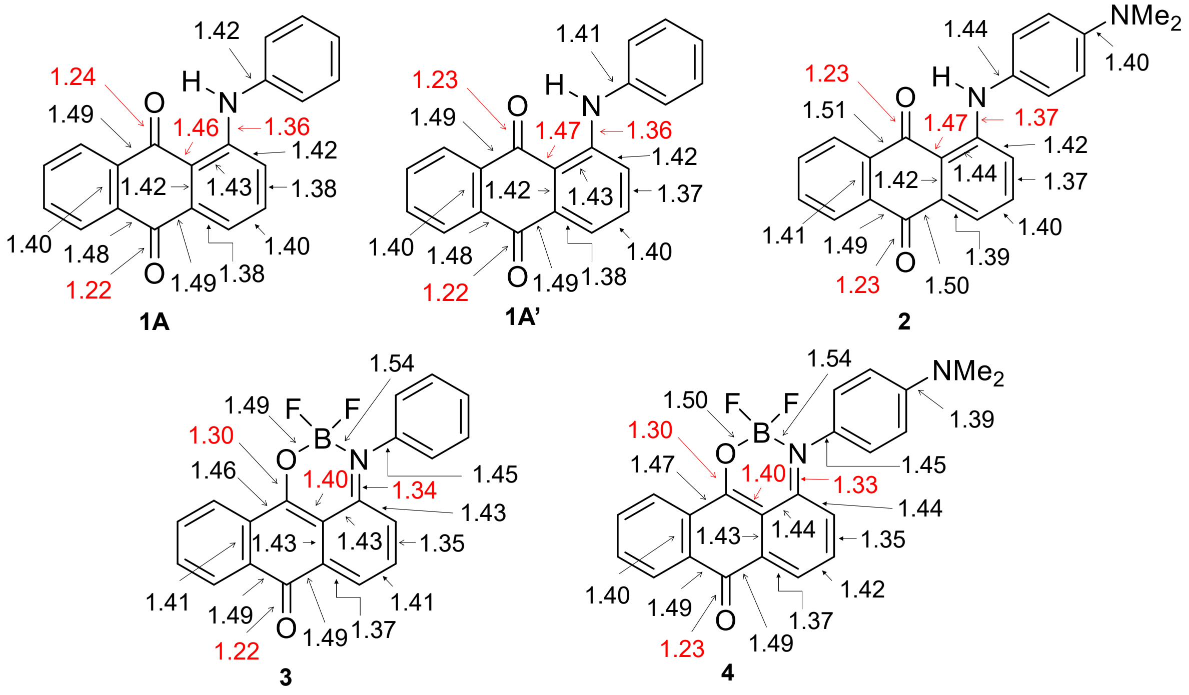

Figure S1. Experimentally obtained bond lengths of **1**–**4**. The unit of bond length is Å.

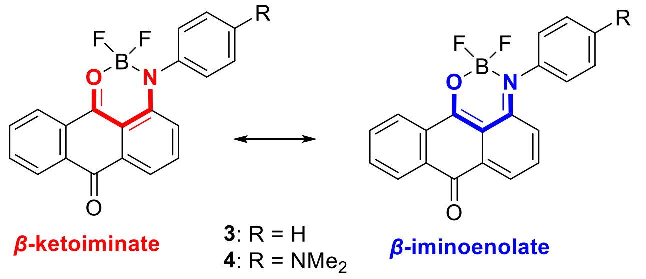

Figure S2. Resonance structures between the β-iminoenolate and β-ketoiminate forms.

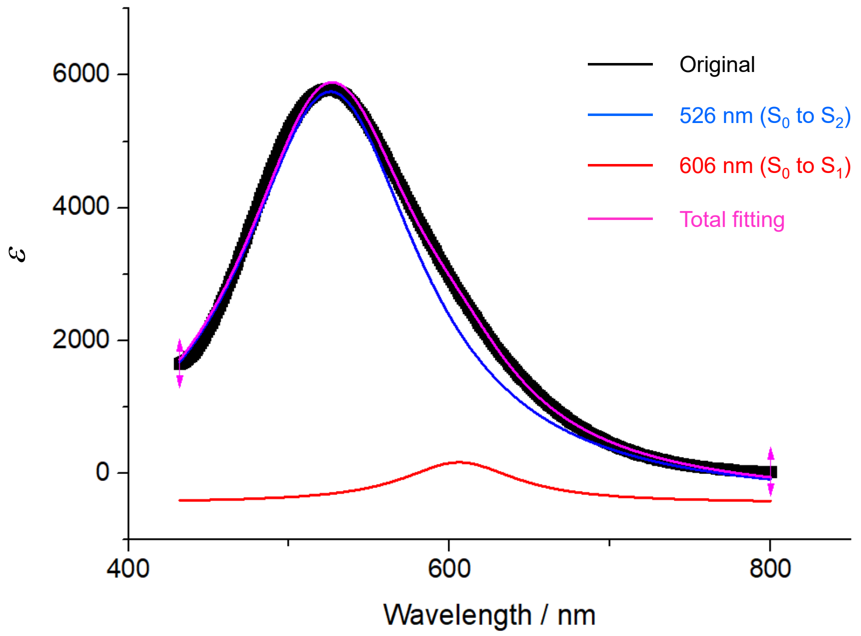

Figure S3. Deconvolution of UV−Vis−NIR absorption spectrum of **2** in dichloromethane.

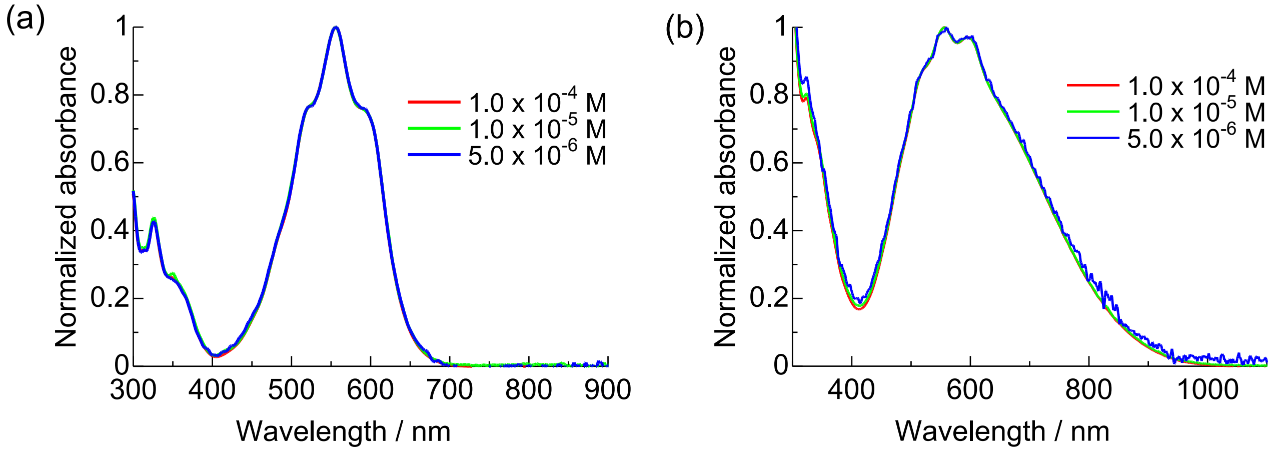

Figure S4. The effect of concentration on the UV−Vis−NIR absorption spectra of (a) **3** and (b) **4** in dichloromethane.

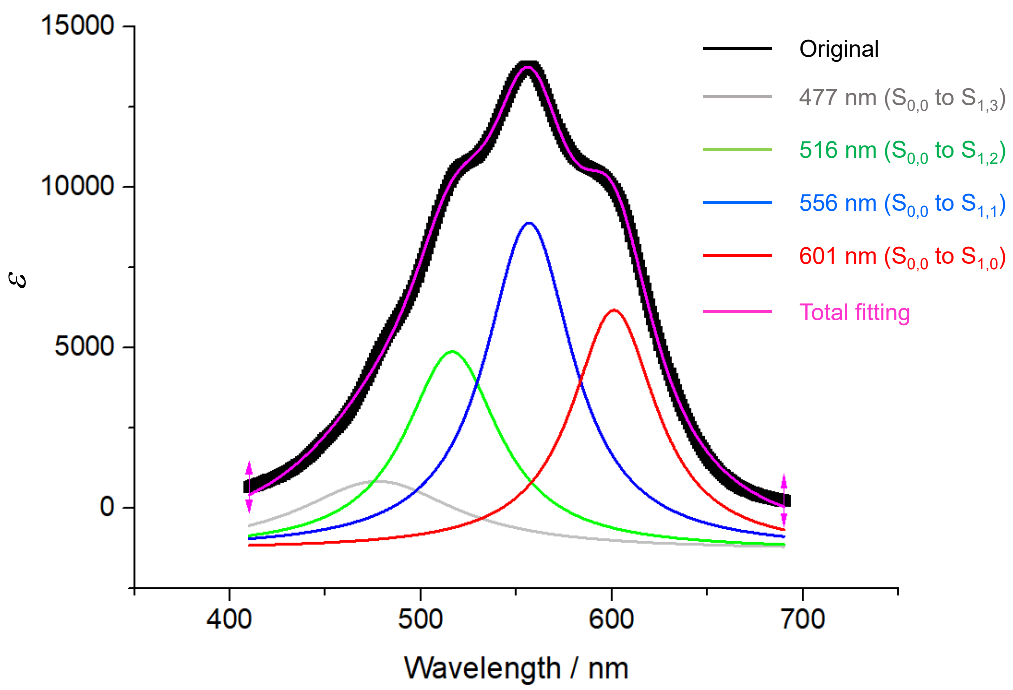

Figure S5. Deconvolution of UV−Vis−NIR absorption spectrum of **3** in dichloromethane.

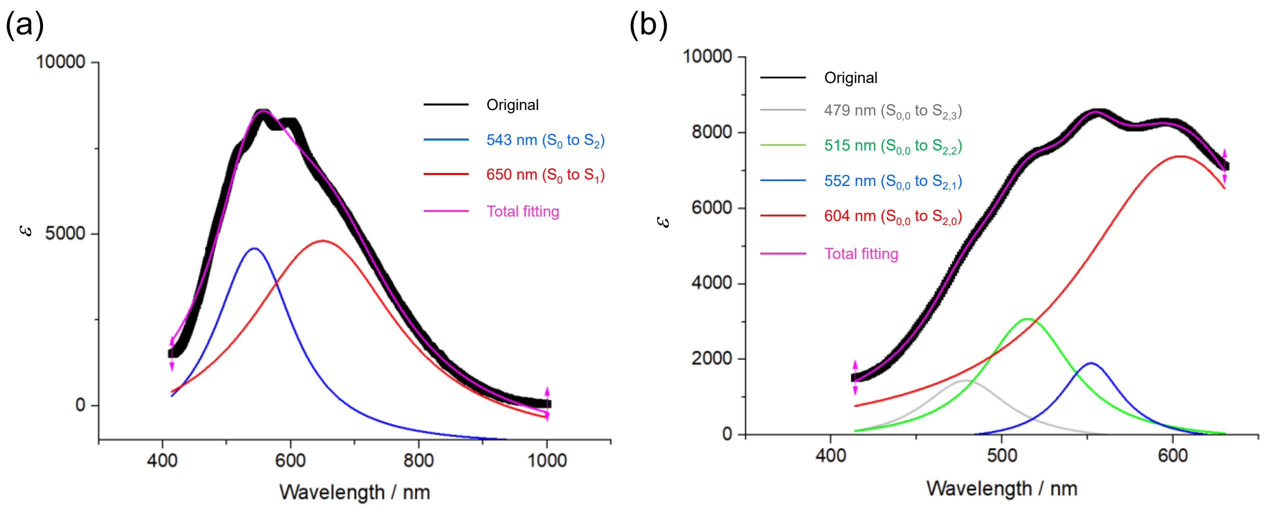

Figure S6. Deconvolution of UV−Vis−NIR absorption spectrum of **4** in dichloromethane: (a) entire region and (b) S_0–S_2 transition region.

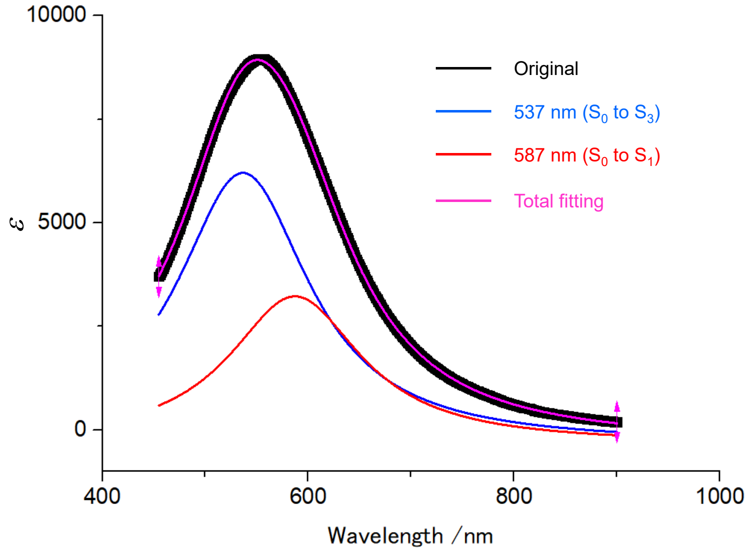

Figure S7. Deconvolution of UV−Vis−NIR absorption spectrum of **6** in dichloromethane.

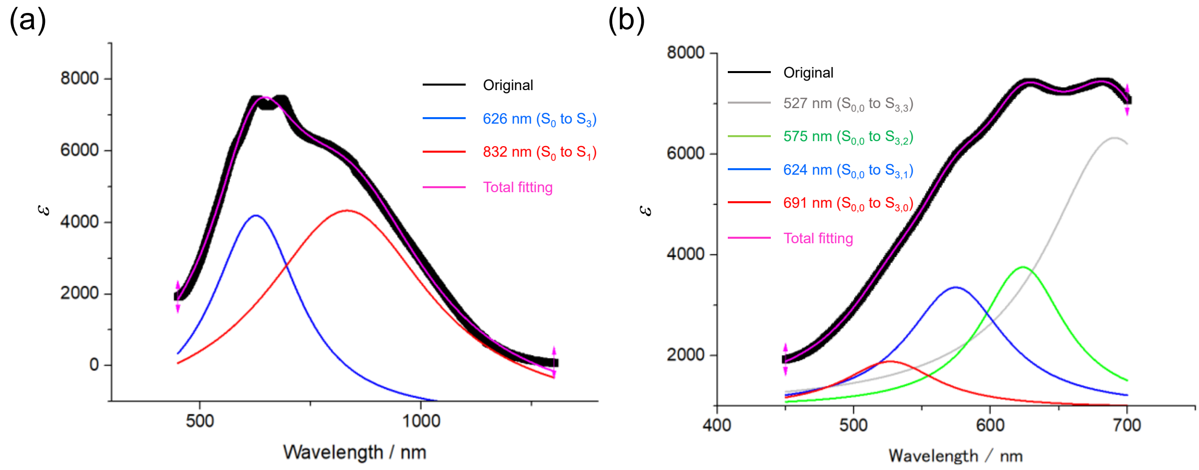

Figure S8. Deconvolution of UV−Vis−NIR absorption spectrum of **8** in dichloromethane: (a) entire region and (b) S_0–S_3 transition region.

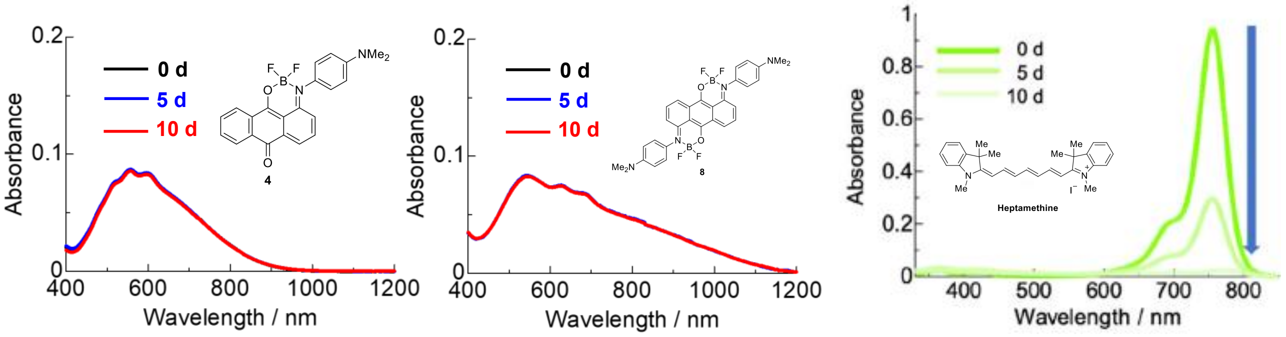

Figure S9. Photostabilities of **4**, **8** and heptamethine cyanine under white light-emitting diode (LED) light (8.5 W, emission system: blue LED + yellow emitting phosphor) in a thermostatic chamber at 25 °C in dichloromethane (**4**: 1 × 10^{-5} M, **8**: 1 × 10^{-5} M, heptamethine cyanine: 1 × 10^{-6} M).

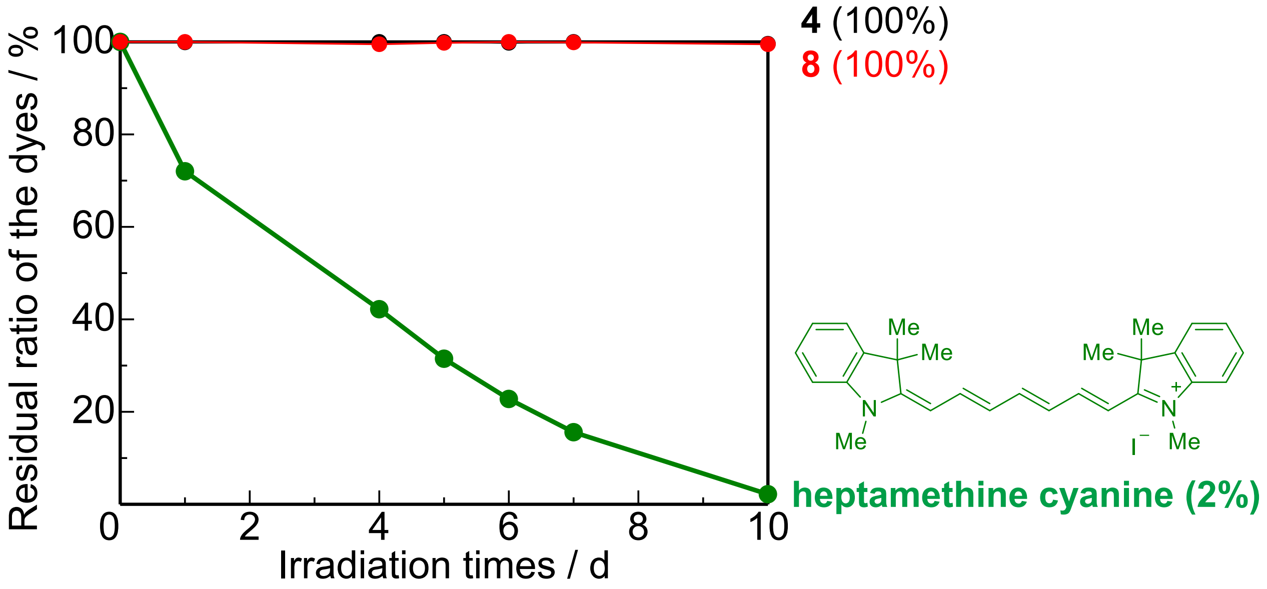

Figure S10. Photostabilities of **4**, **8** and heptamethine cyanine under white LED light. The dye residuals were calculated from the changes in absorbance at the λ_{max} in the UV–Vis–NIR spectra.

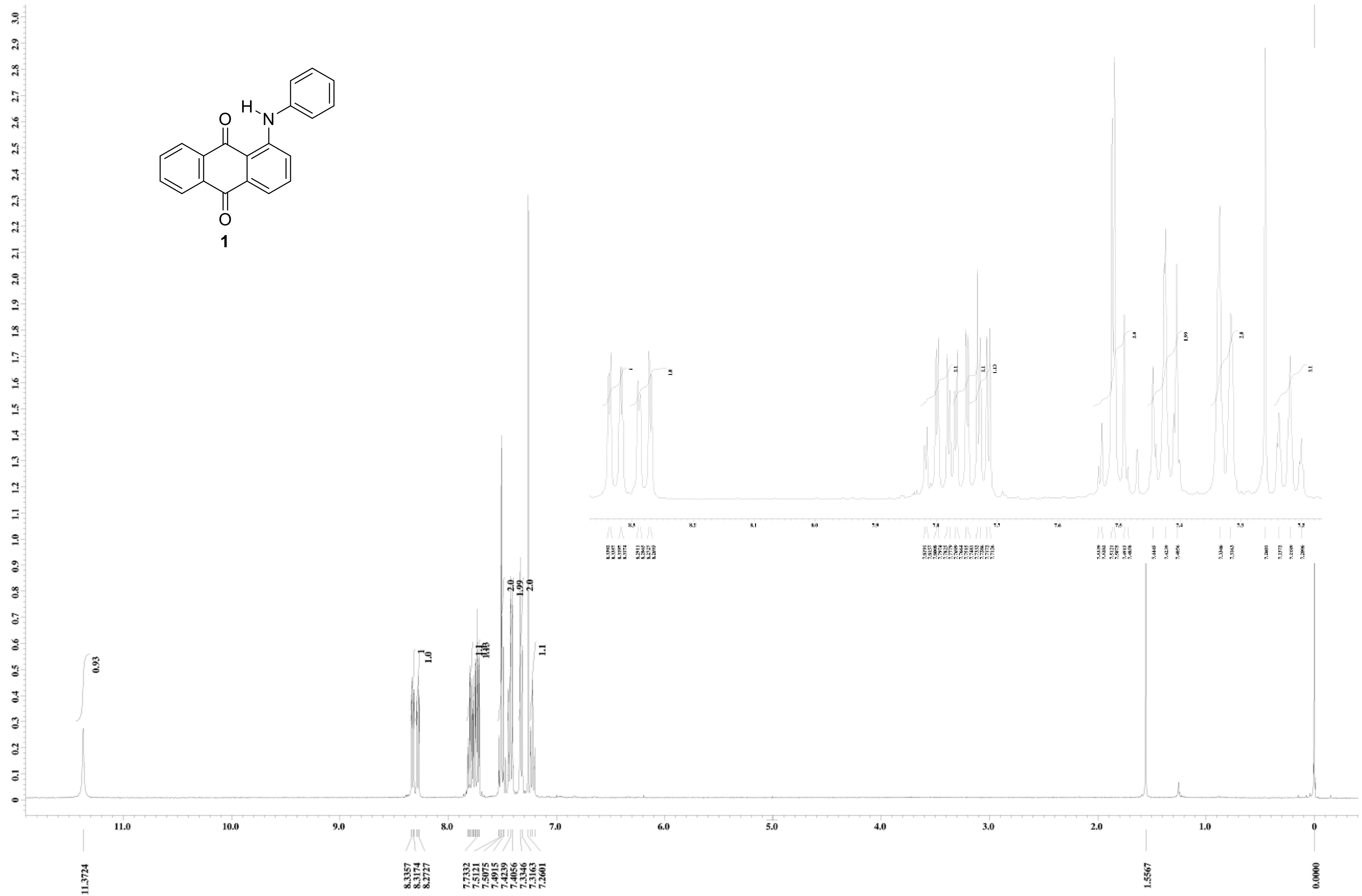

Figure S11. ^{1}H NMR spectrum of **1** (400 MHz, $CDCl_3$).

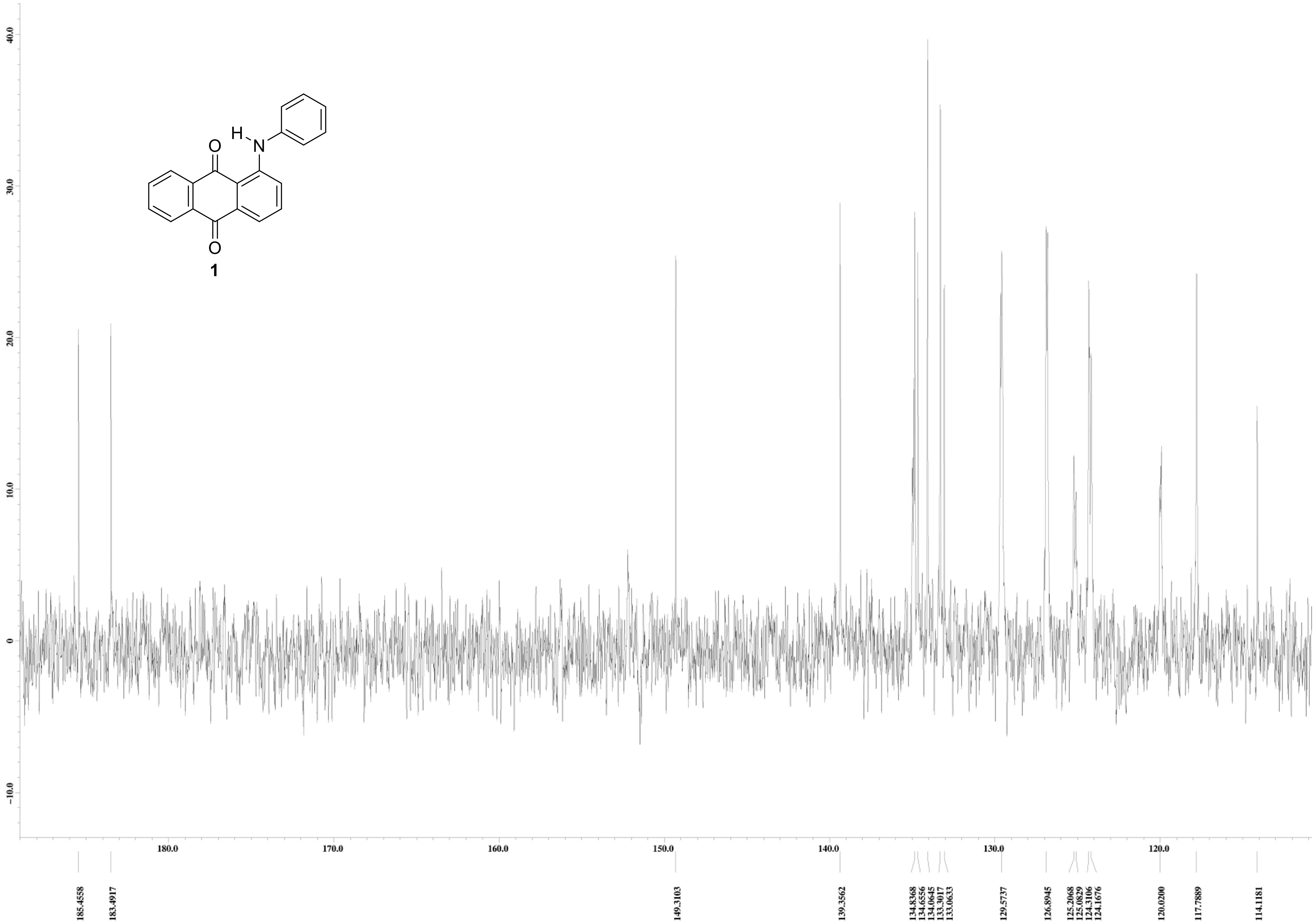

Figure S12. ^{13}C NMR spectrum of **1** (100 MHz, $CDCl_3$).

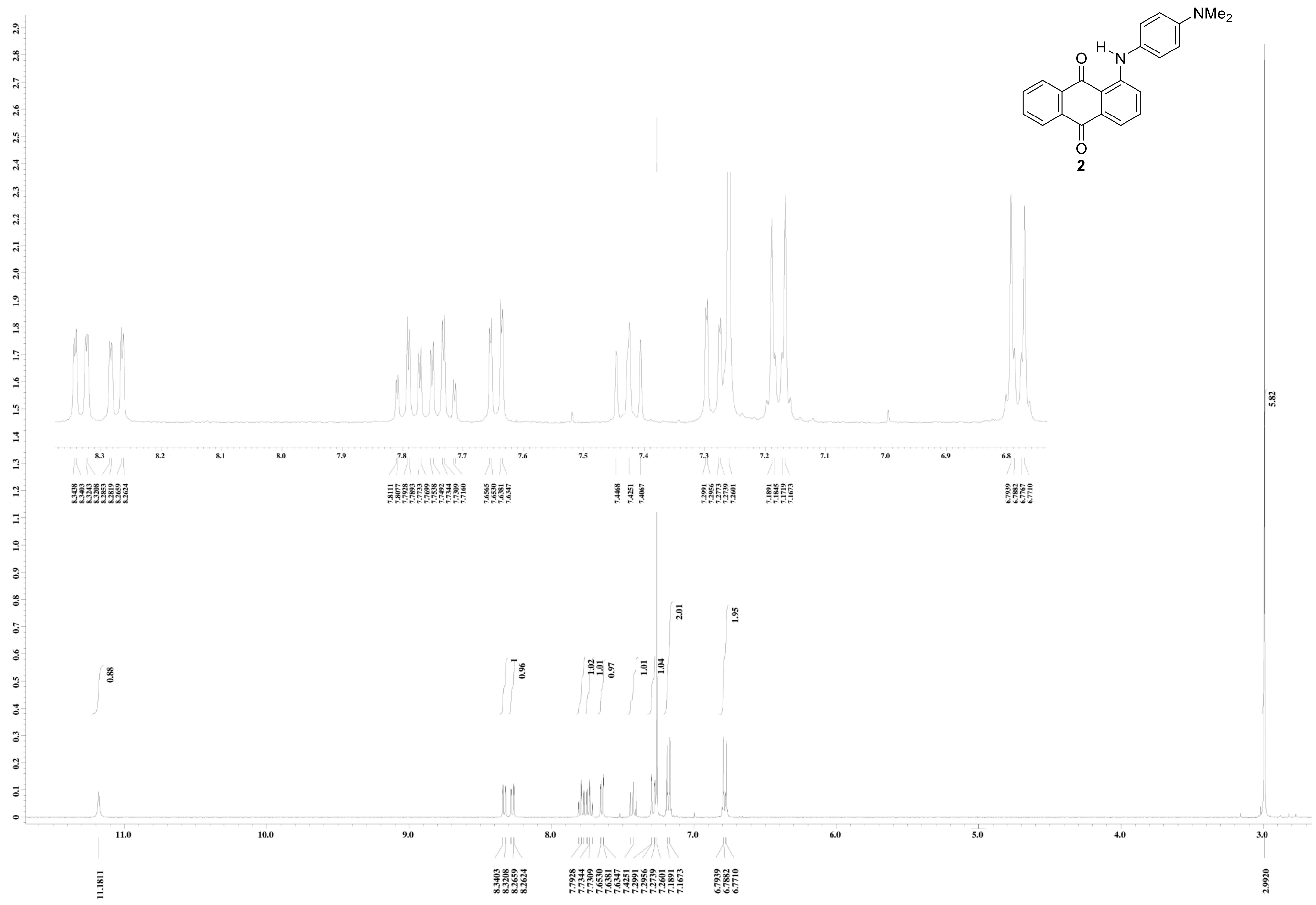

Figure S13. ^{1}H NMR spectrum of **2** (400 MHz, CDCl$_3$).

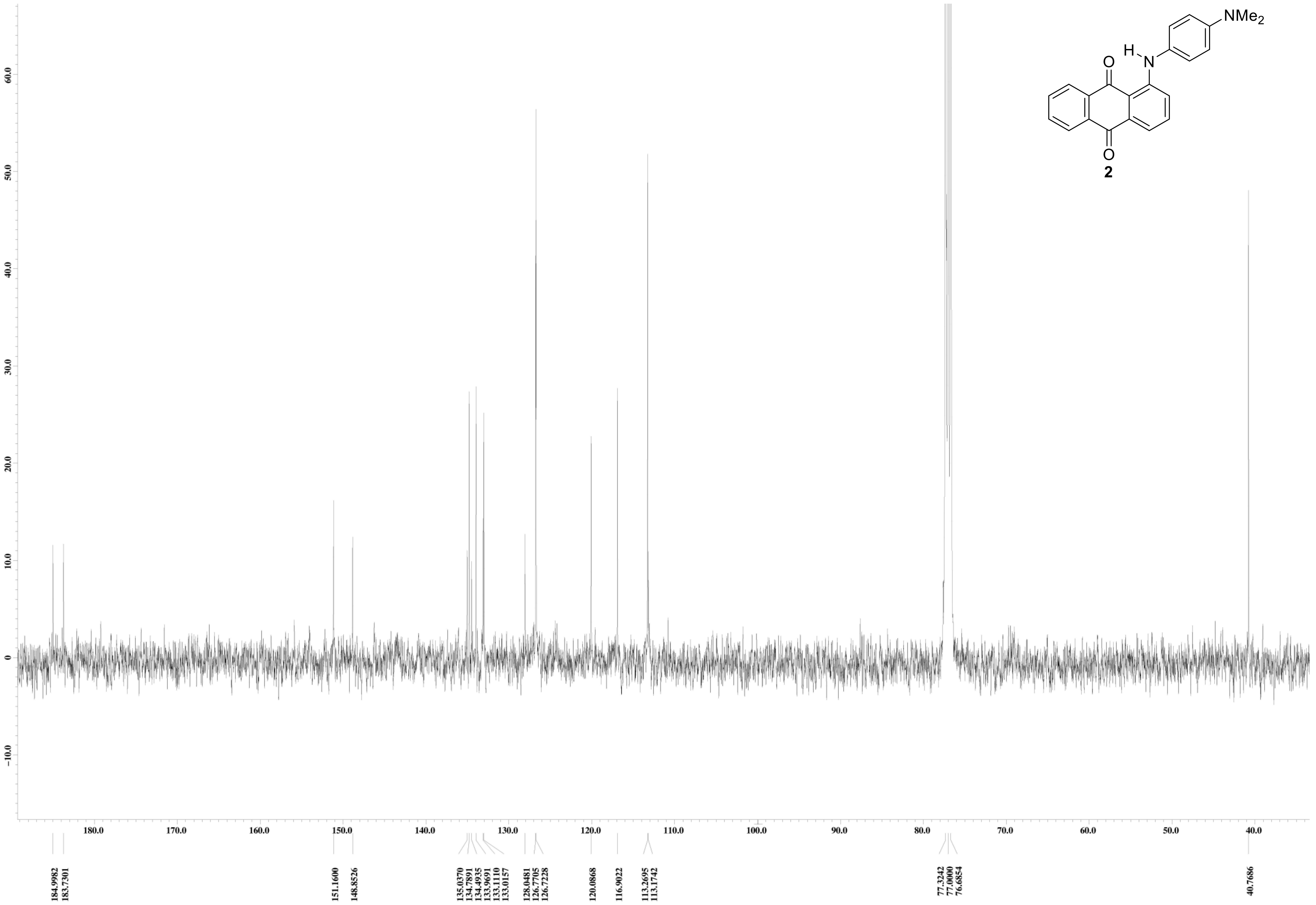

Figure S14. ^{13}C NMR spectrum of **2** (100 MHz, $CDCl_3$).

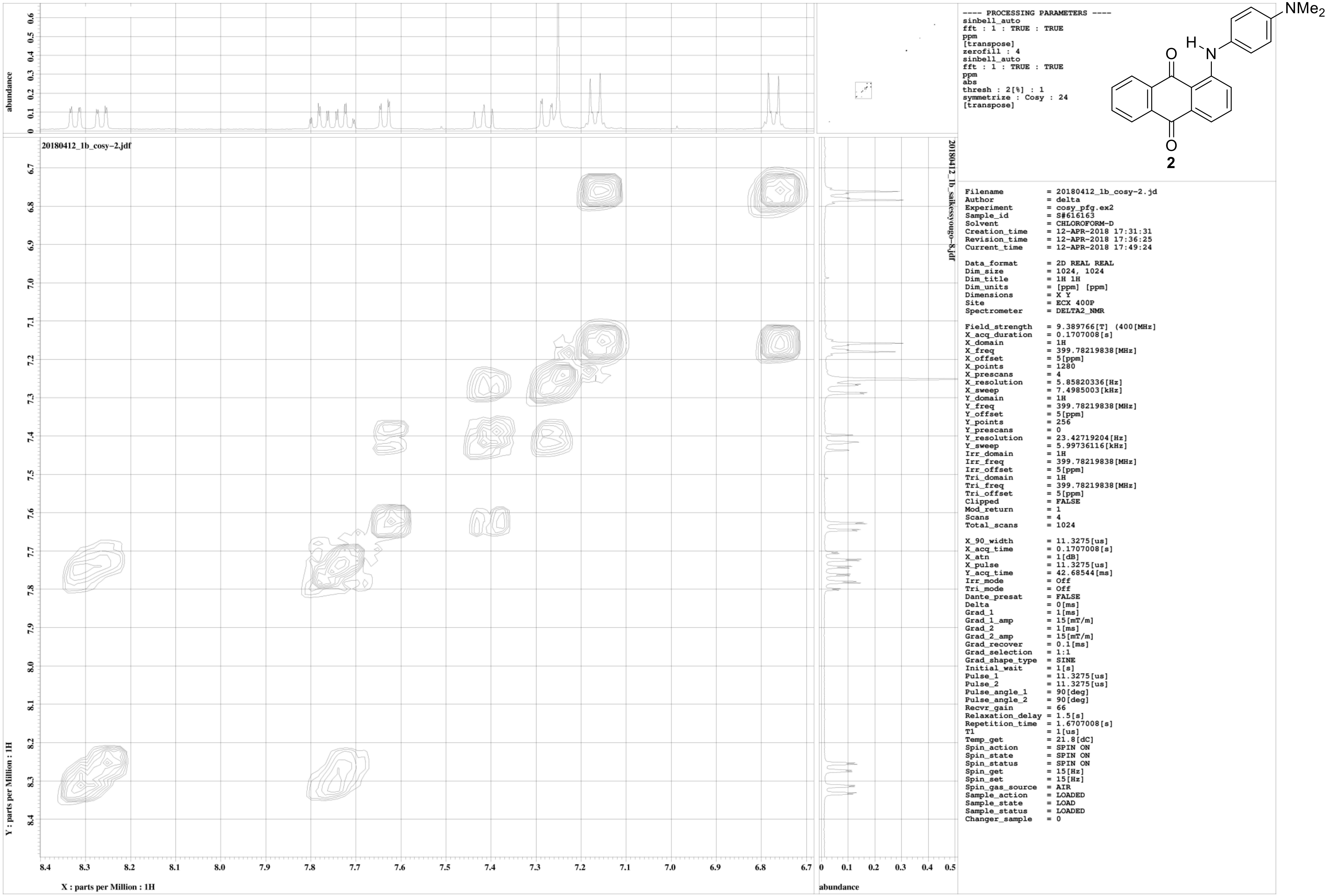

Figure S15. COSY spectrum of **2**.

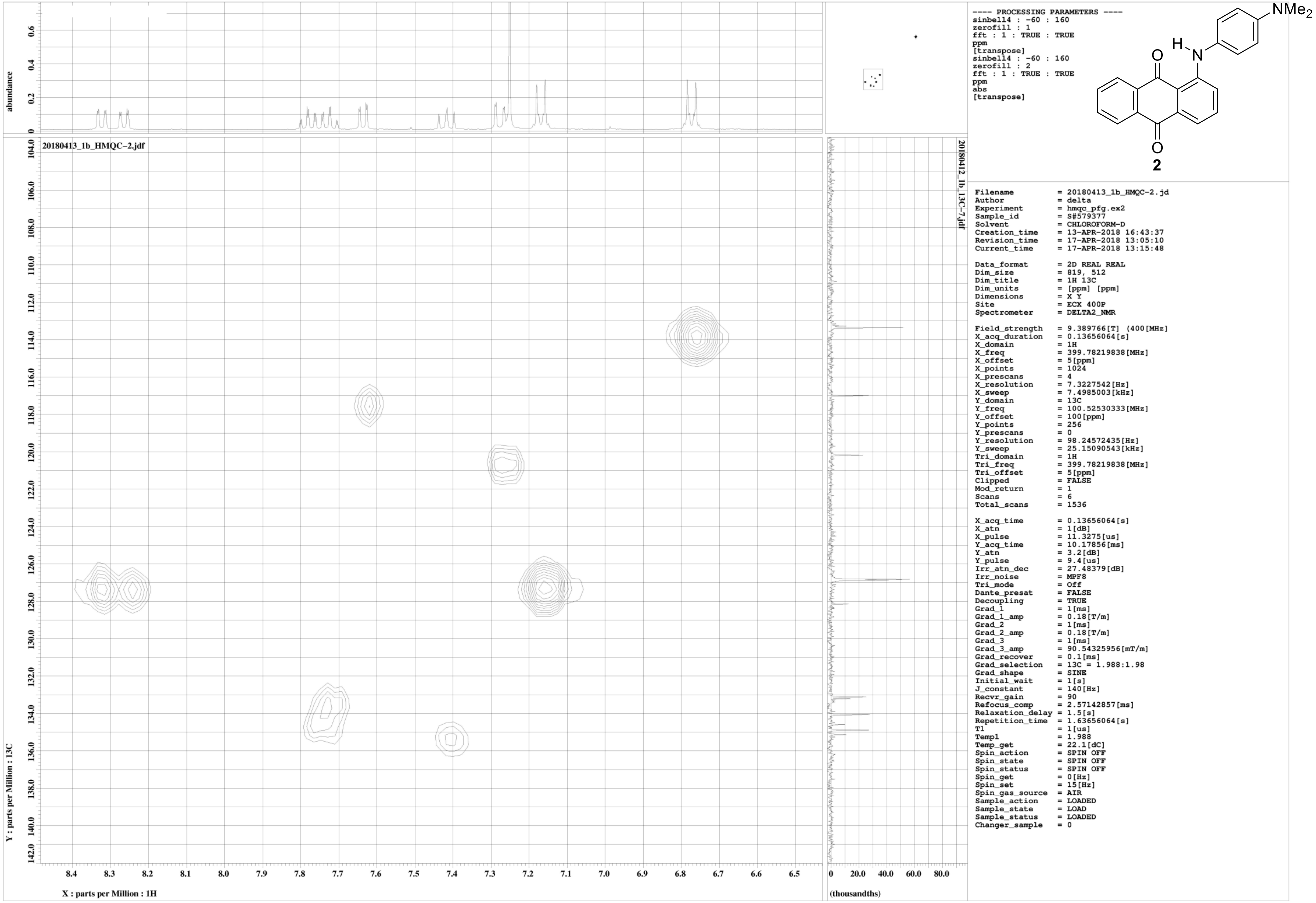

Figure S16. HMQC spectrum of **2**.

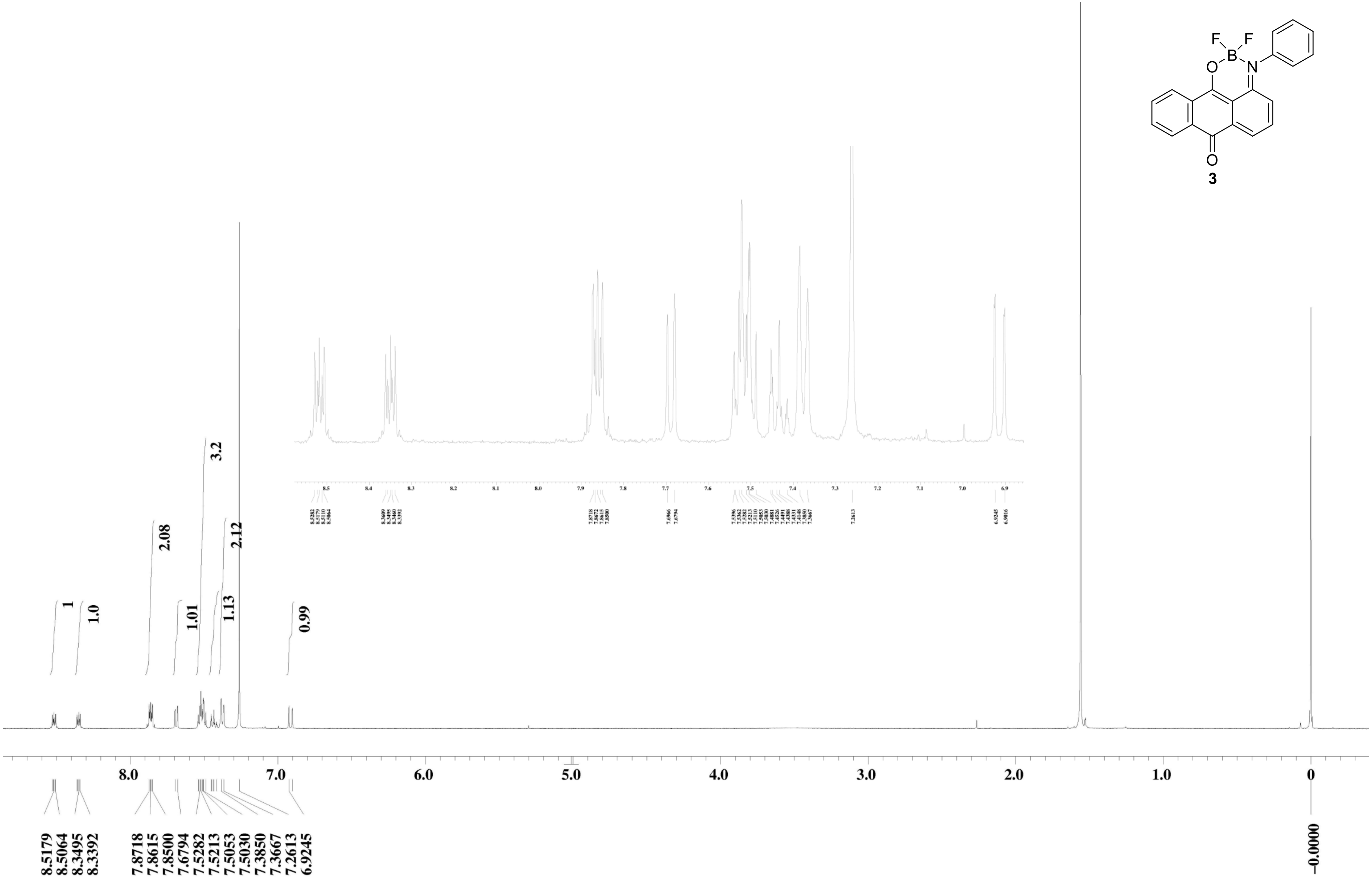

Figure S17. ^{1}H NMR spectrum of **3** (400 MHz, $CDCl_3$).

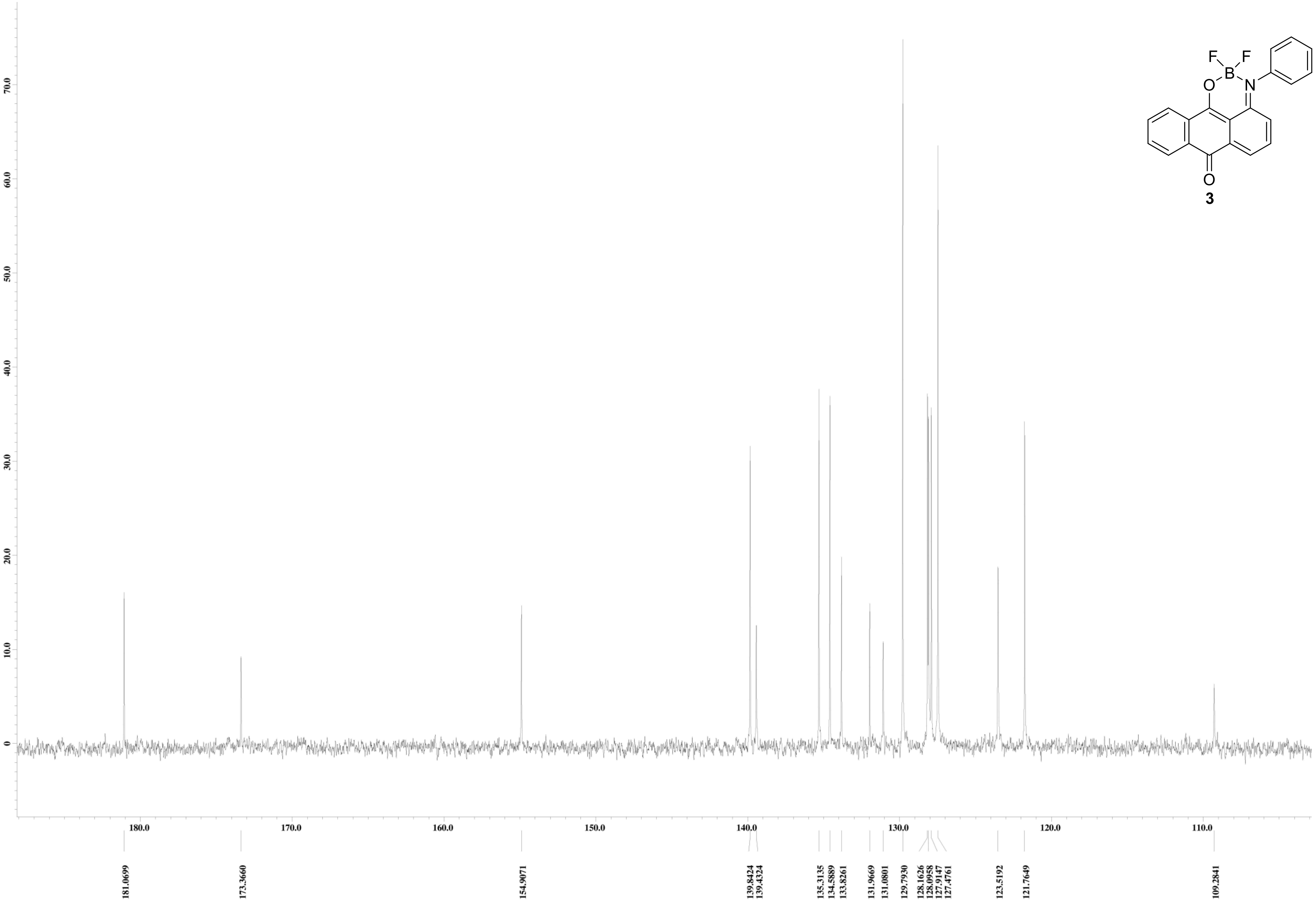

Figure S18. ^{13}C NMR spectrum of **3** (100 MHz, $CDCl_3$).

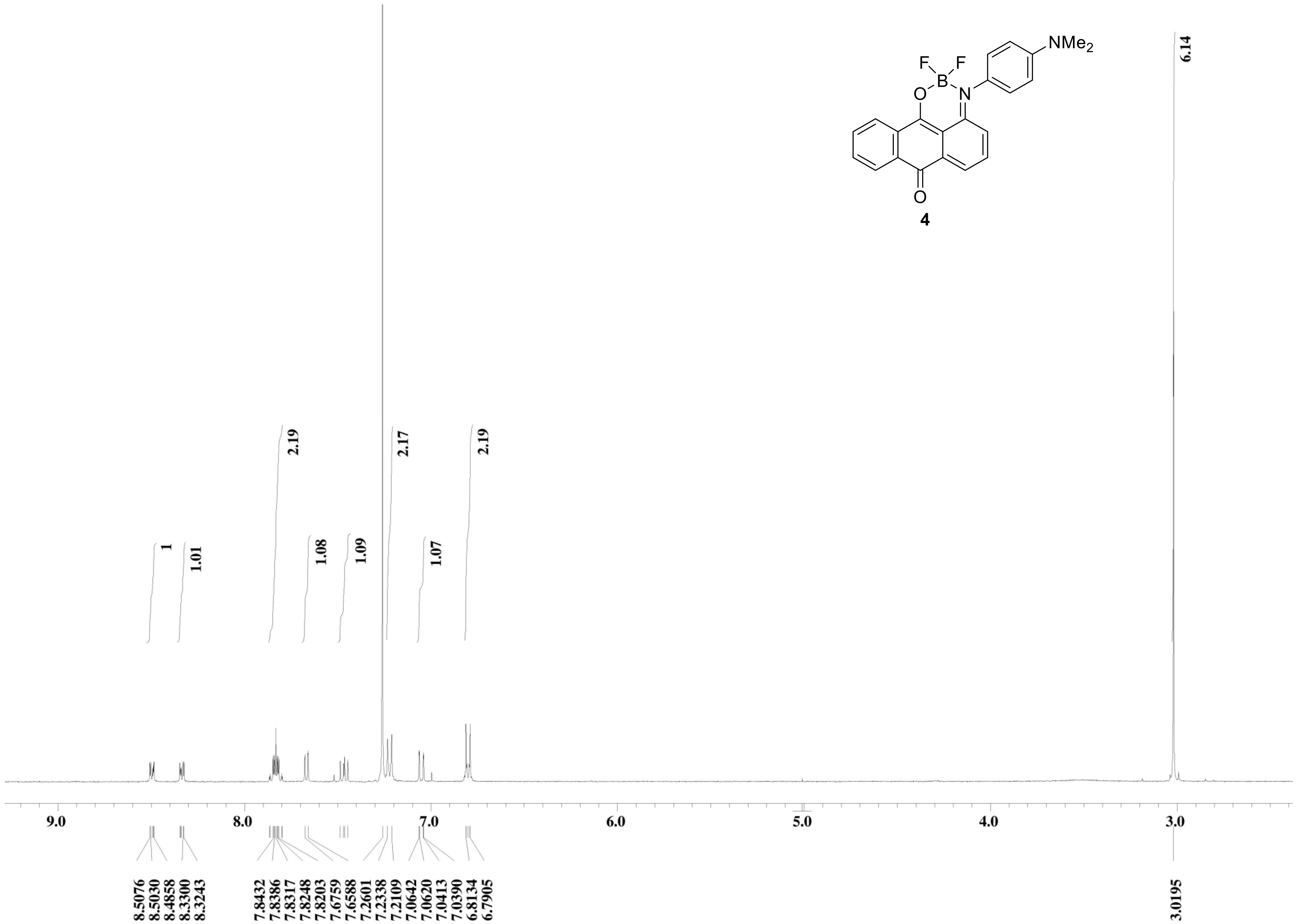

Figure S19. ^{1}H NMR spectrum of **4** (400 MHz, $CDCl_3$).

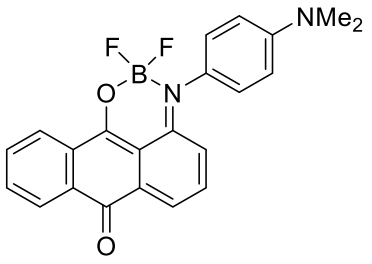

4

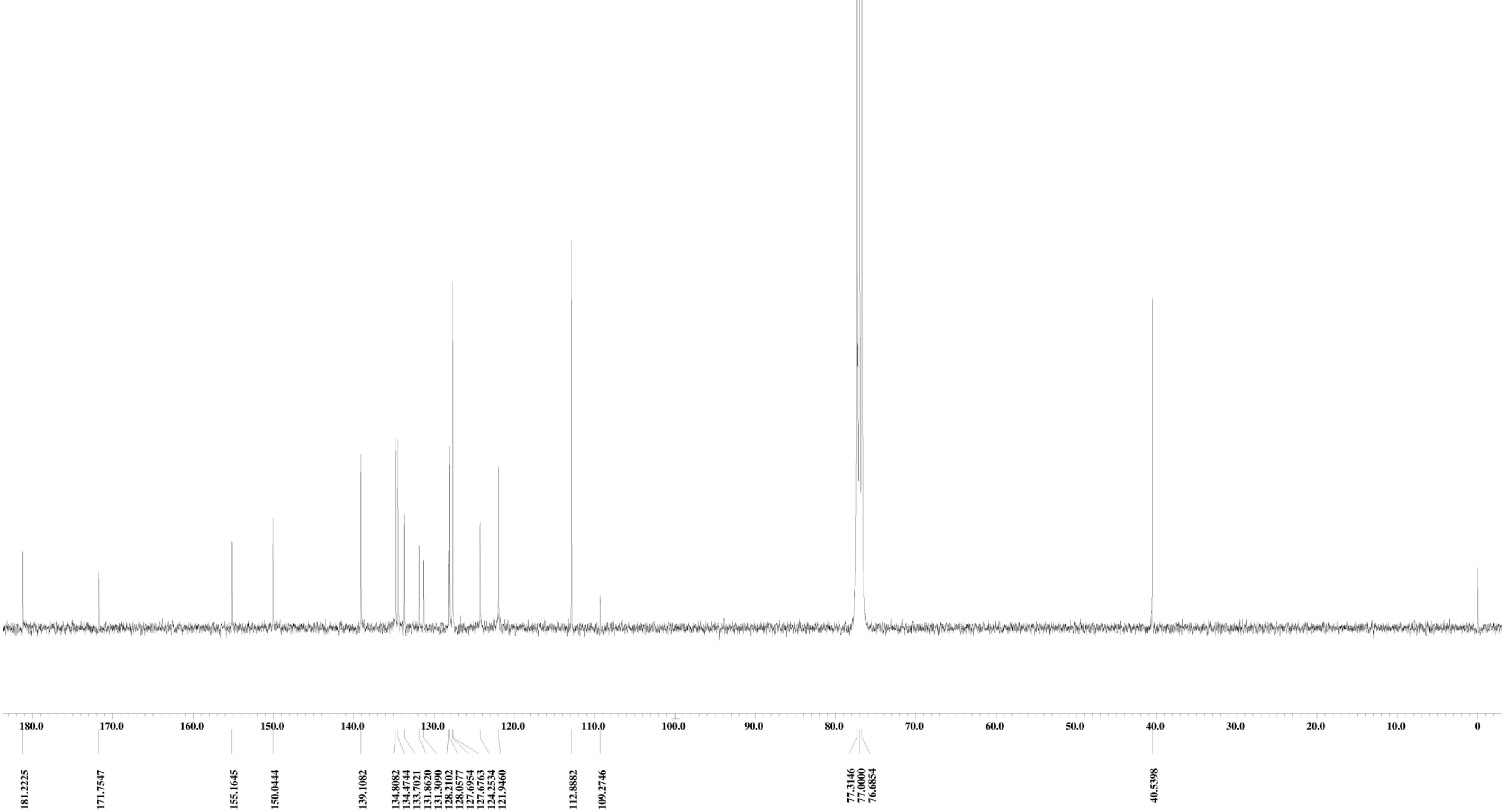

Figure S20. ^{13}C NMR spectrum of **4** (100 MHz, $CDCl_3$).

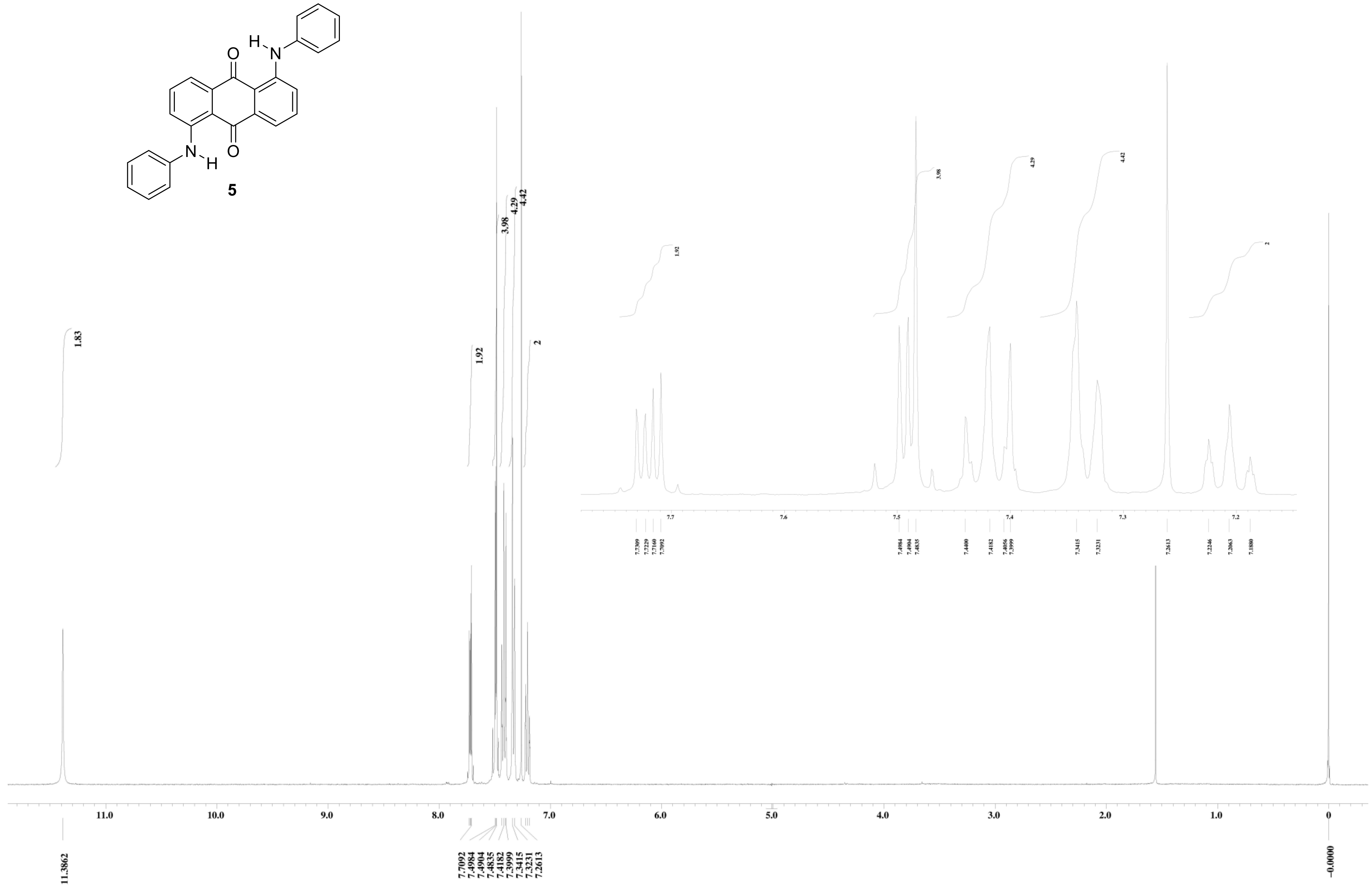

Figure S21. ^{1}H NMR spectrum of **5** (400 MHz, $CDCl_3$).

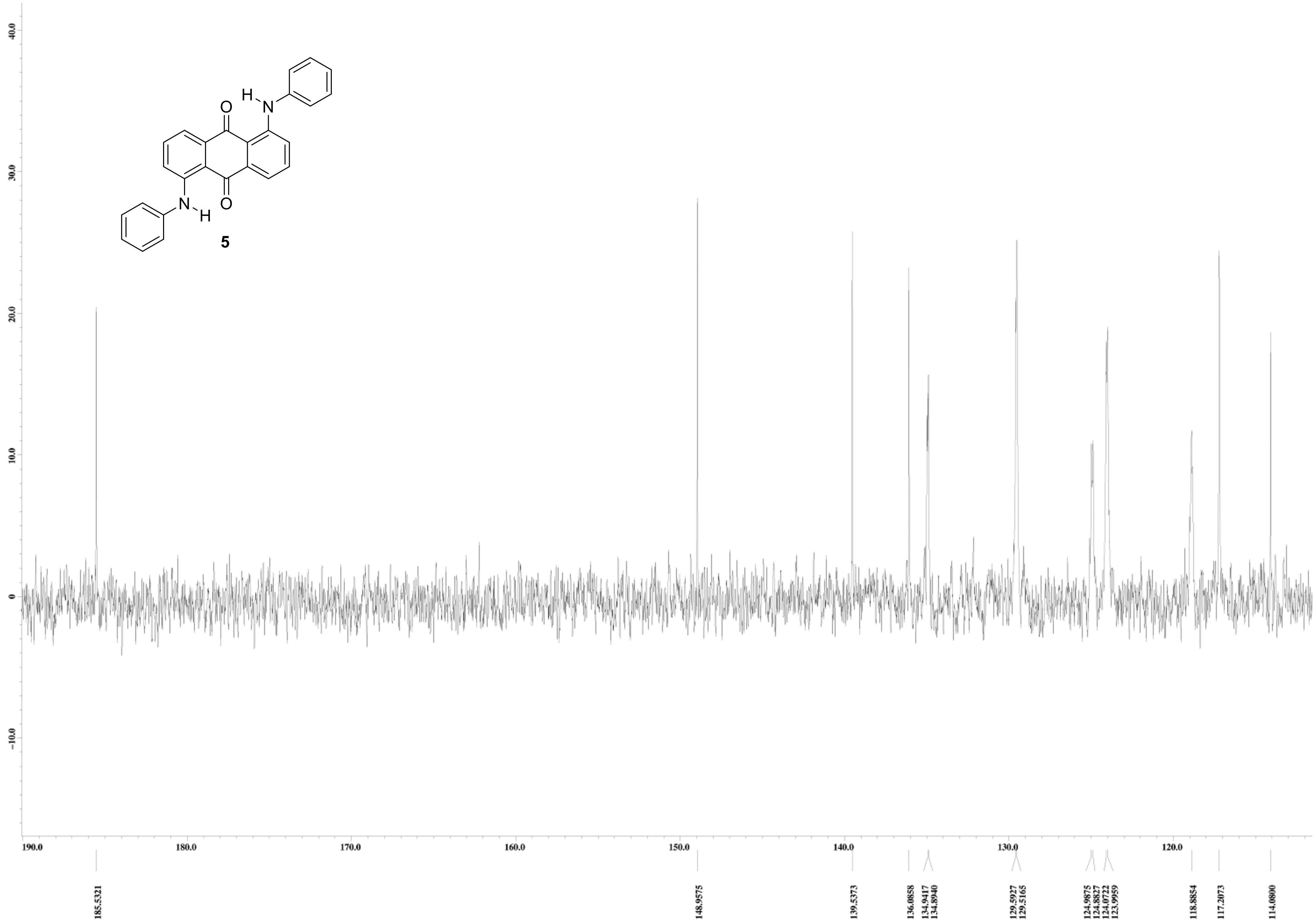

Figure S22. ^{13}C NMR spectrum of **5** (100 MHz, $CDCl_3$).

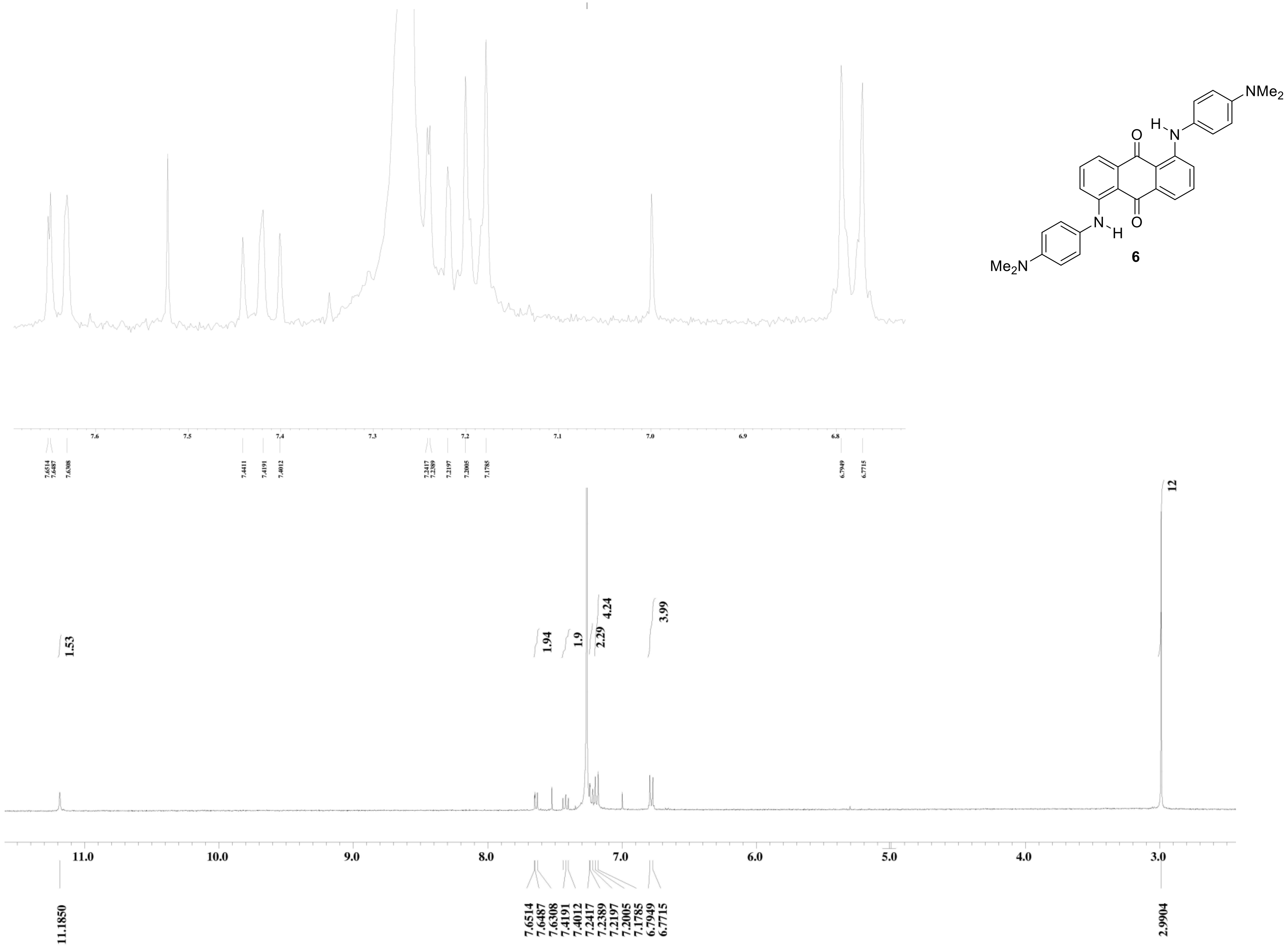

Figure S23. ^1H NMR spectrum of **6** (400 MHz, $CDCl_3$).

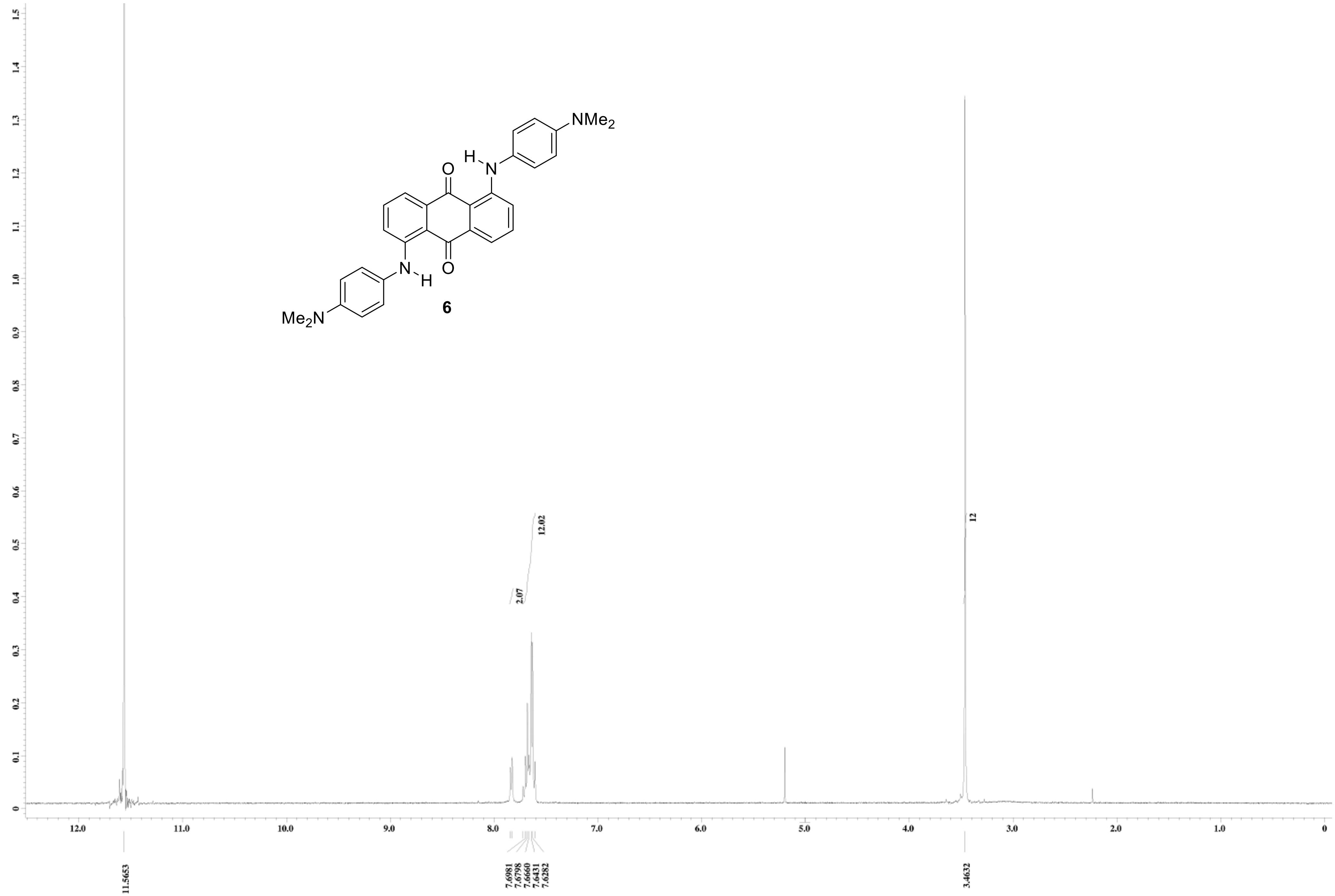

Figure S24. ^{1}H NMR spectrum of **6** (400 MHz, TFA-*d*).

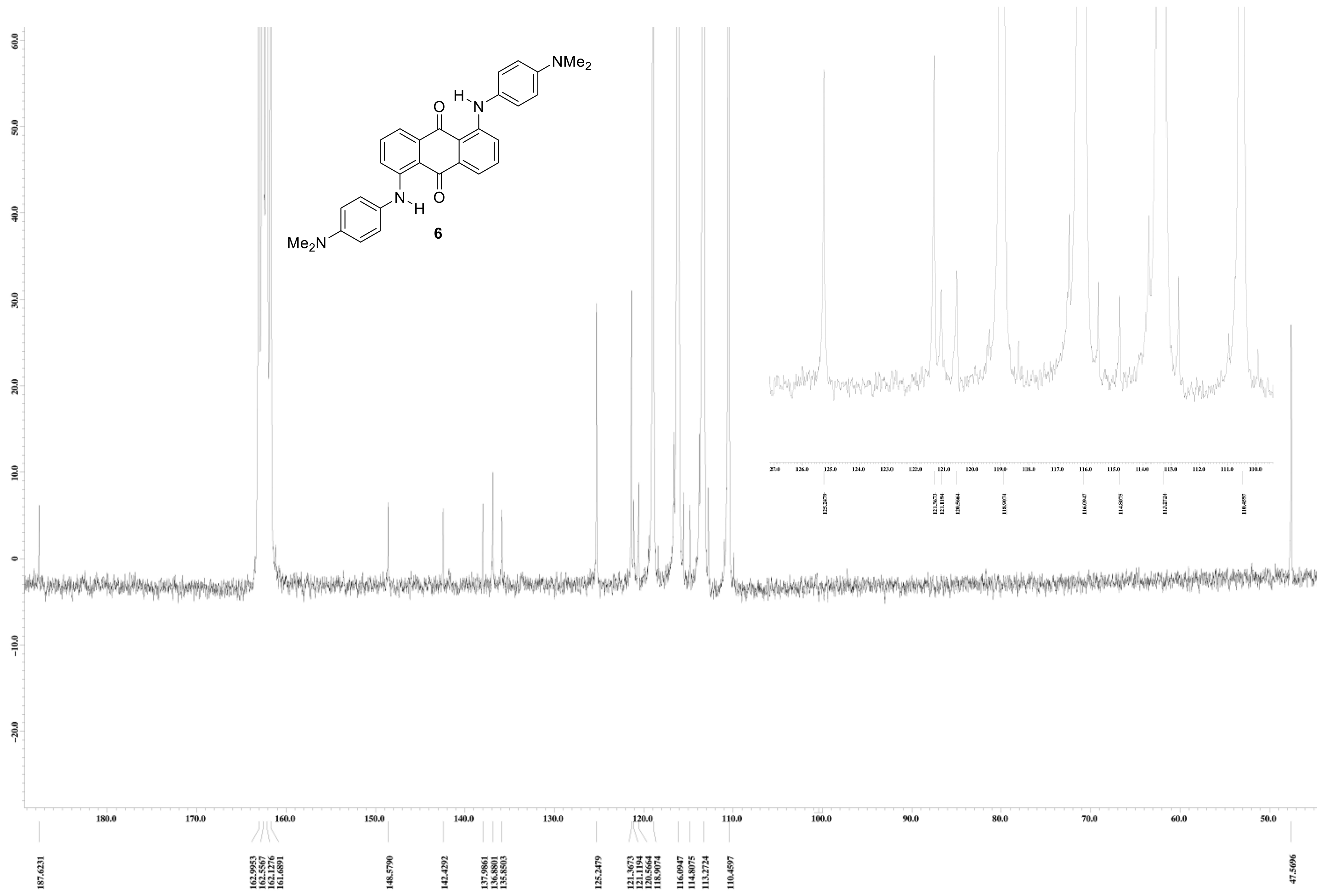

Figure S25. ^{13}C NMR spectrum of **6** (100 MHz, TFA-*d*).

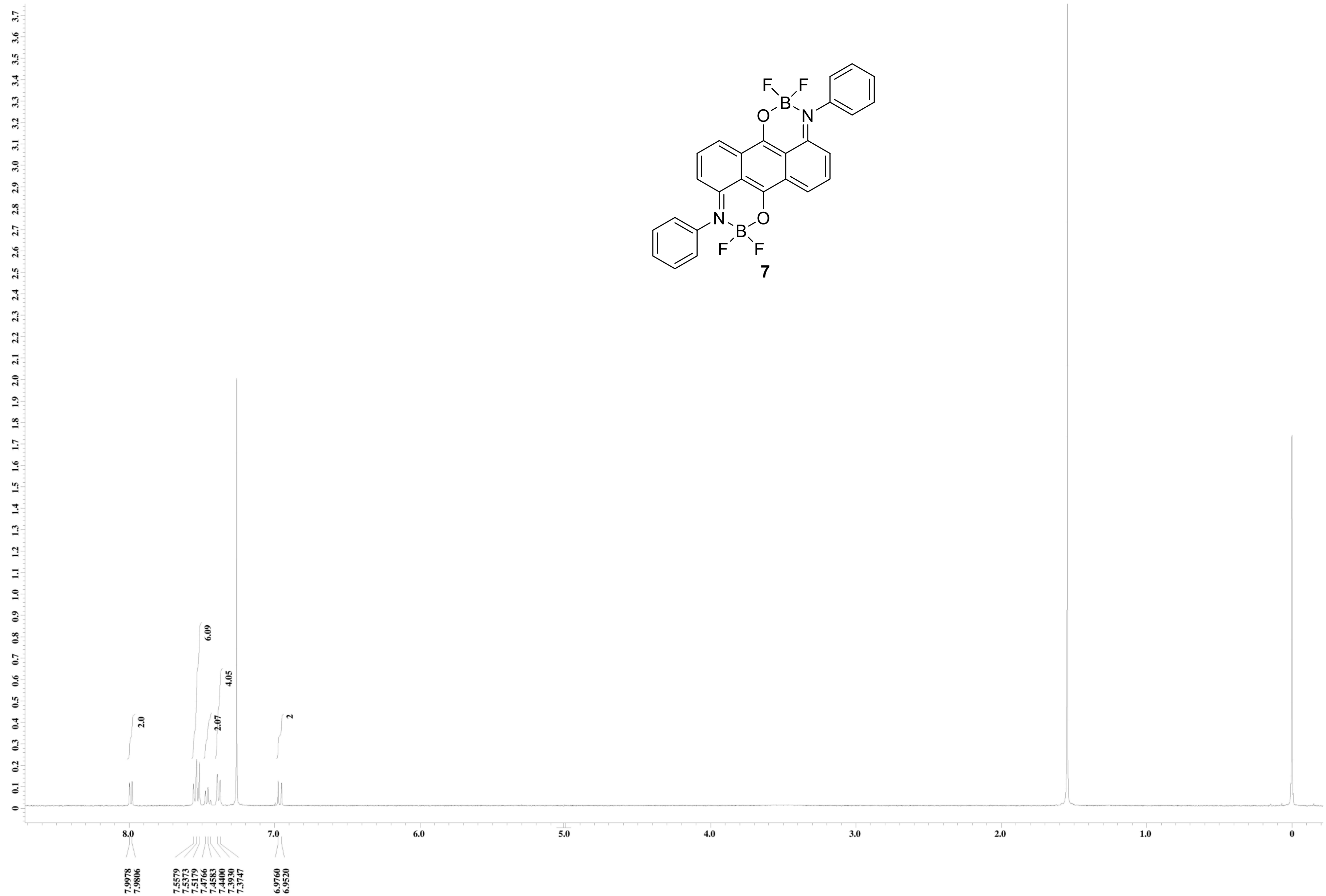

Figure S26. ^{1}H NMR spectrum of **7** (400 MHz, $CDCl_3$).

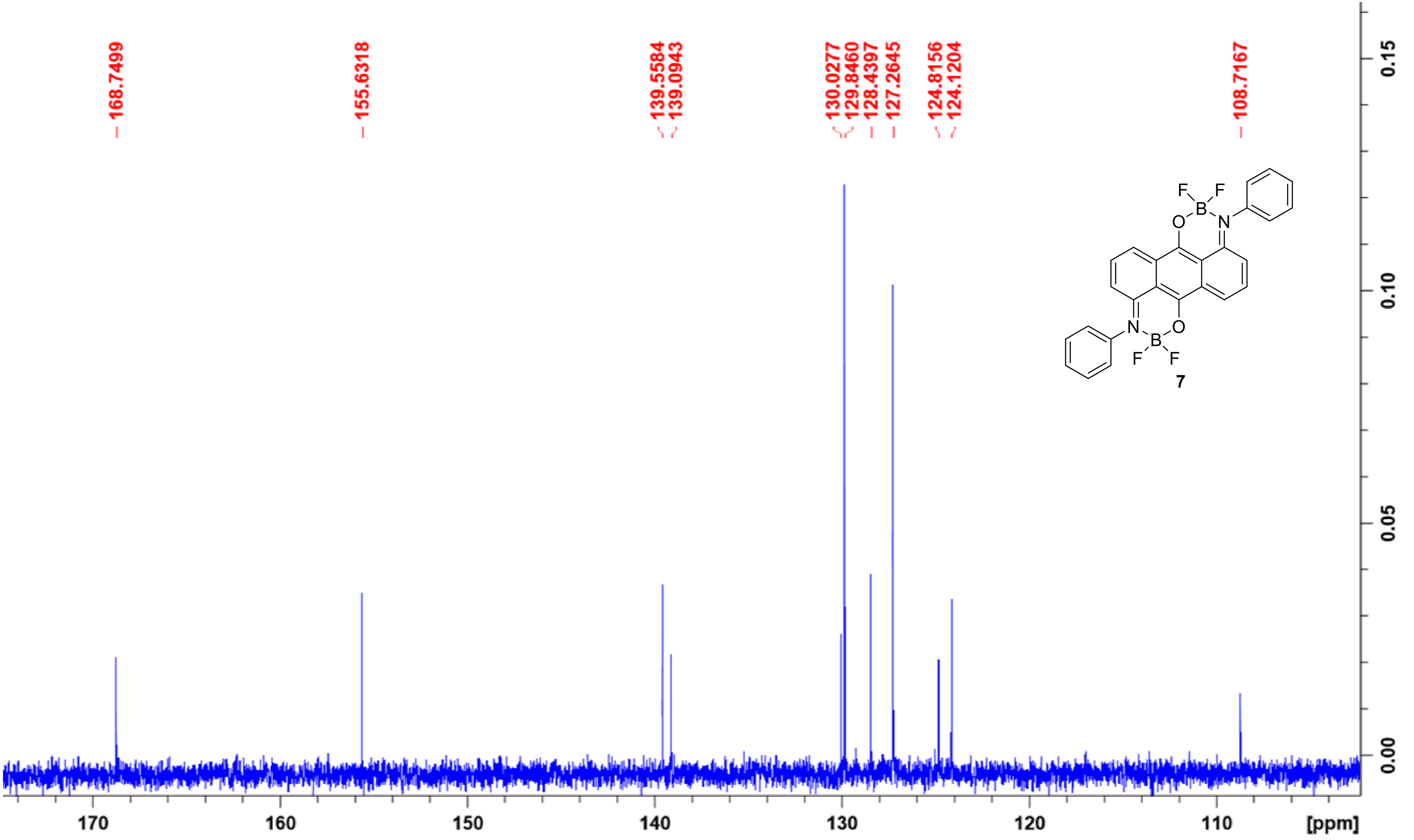

Figure S27. ^{13}C NMR spectrum of **7** (200 MHz, $CDCl_3$).

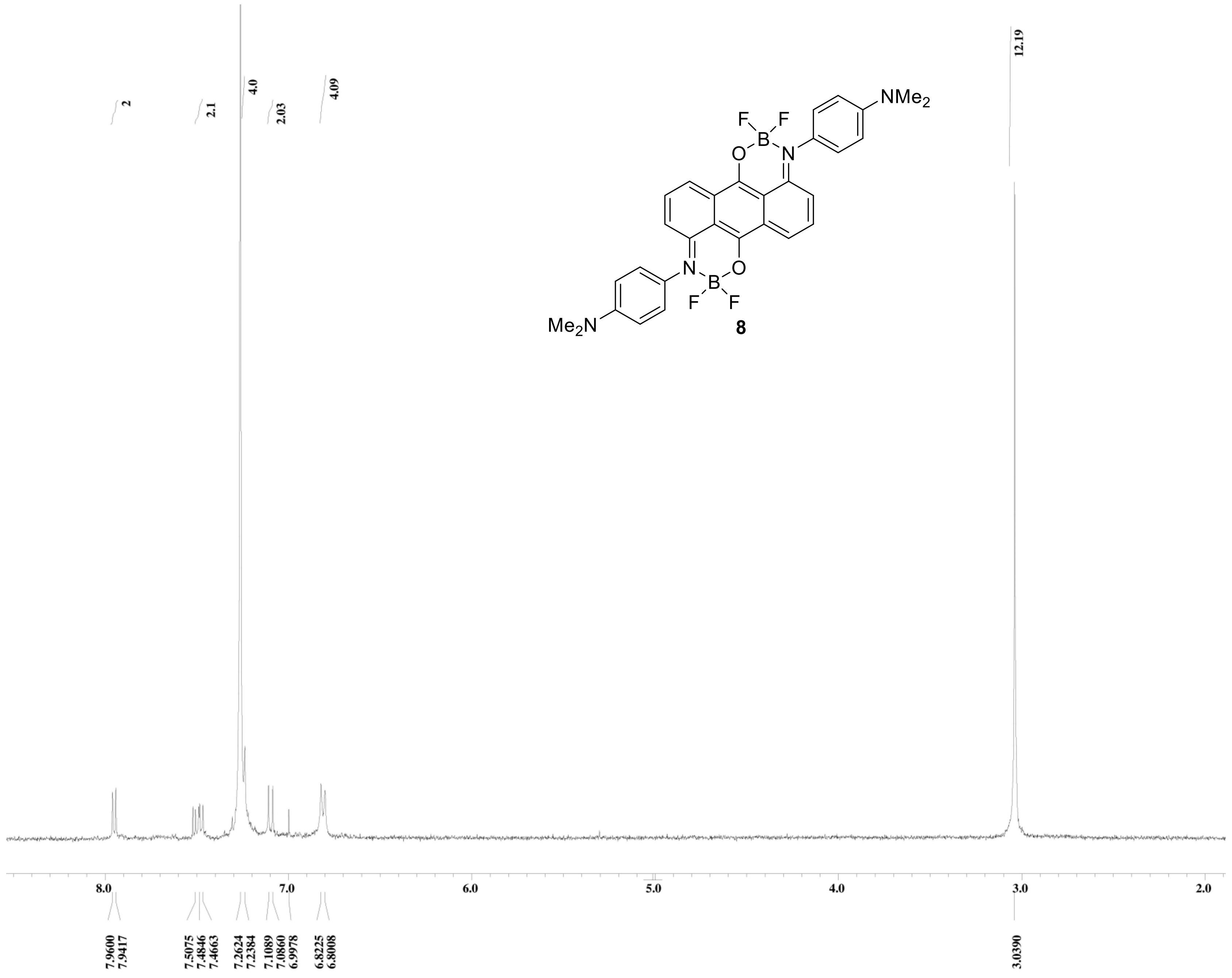

Figure S28. ^{1}H NMR spectrum of **8** (400 MHz, $CDCl_3$).

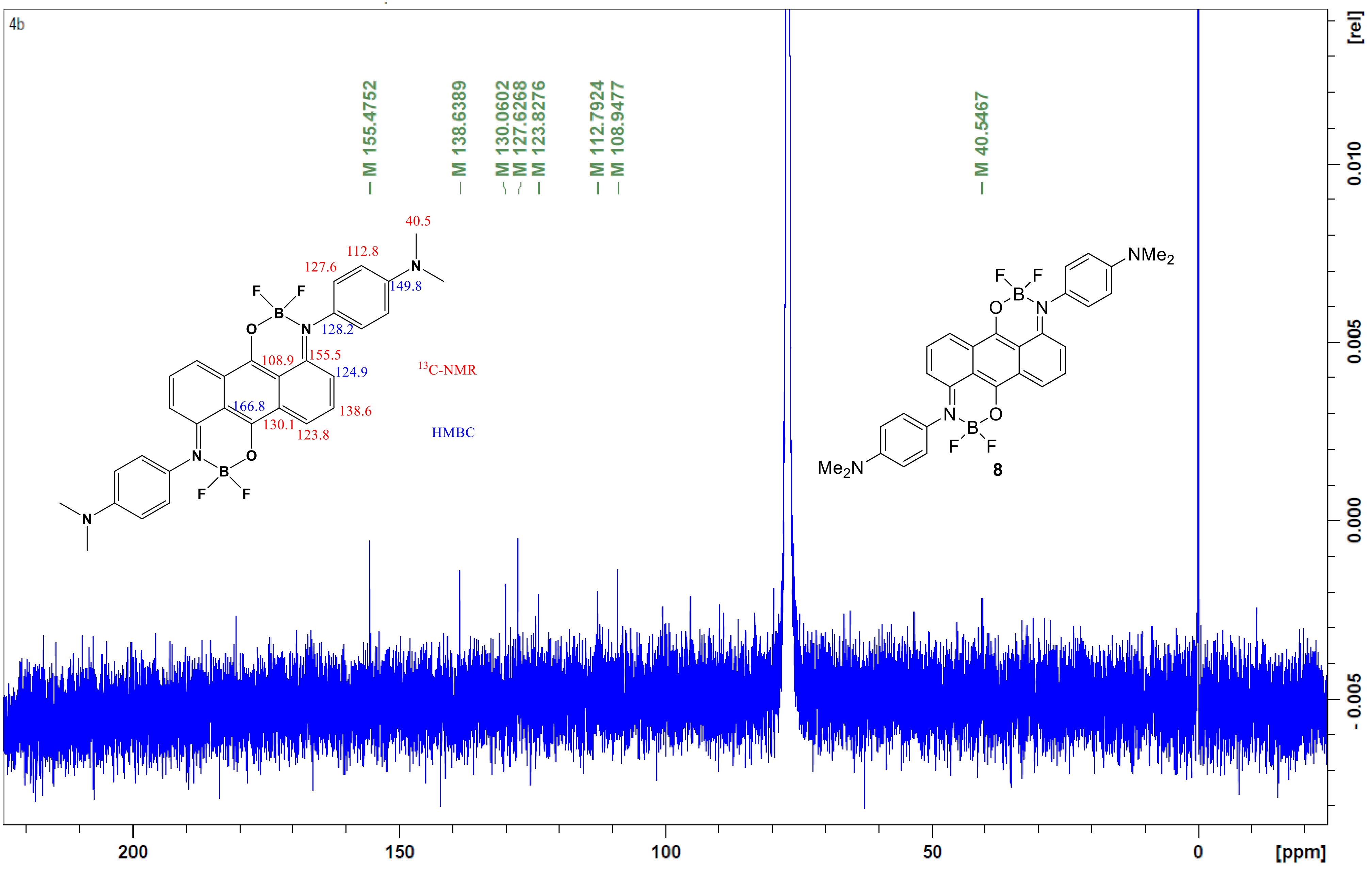

Figure S29. ^{13}C NMR spectrum of **8** (200 MHz, $CDCl_3$).

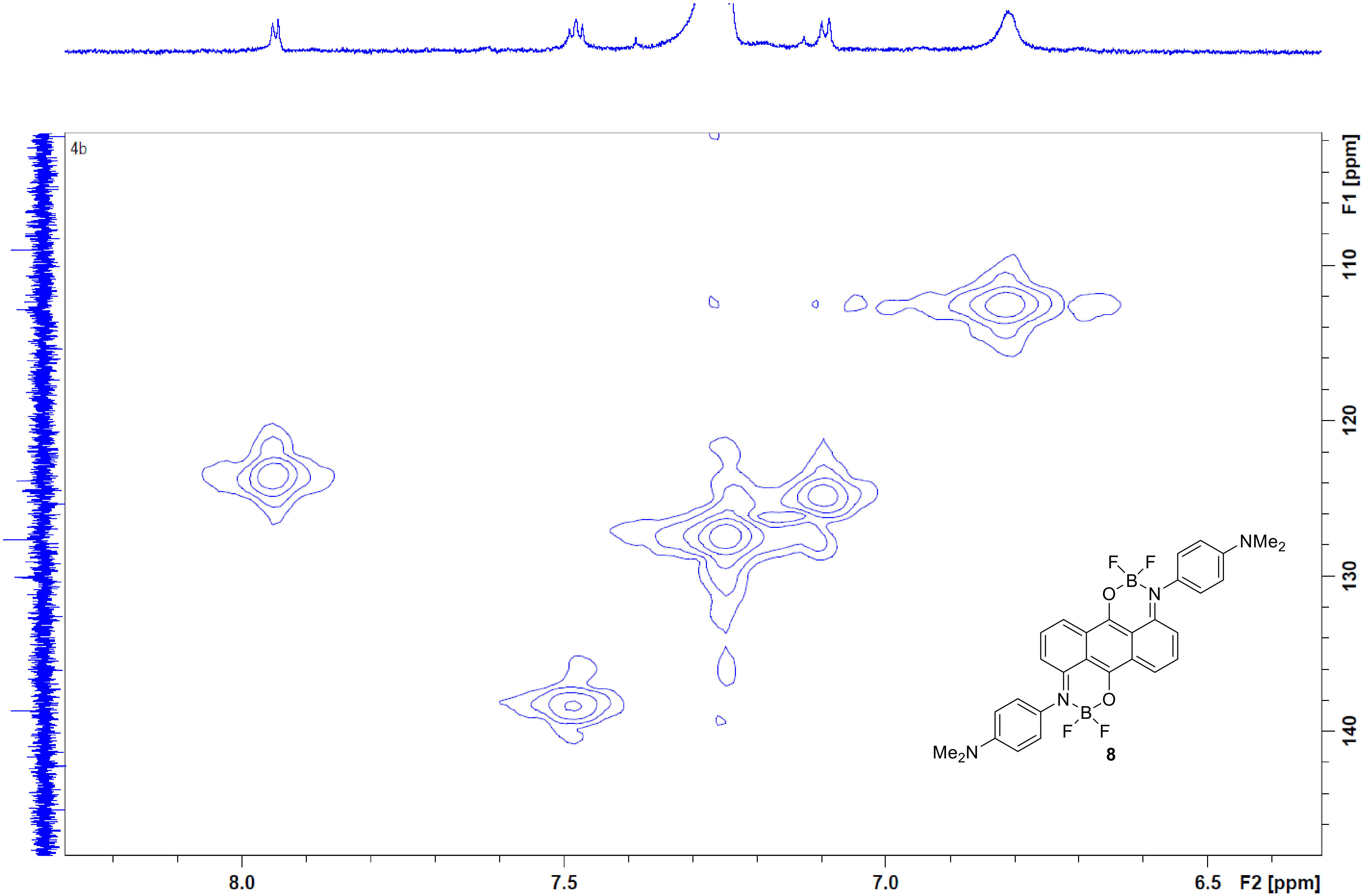

Figure S30. HMQC spectrum of **8** (800 MHz, $CDCl_3$).

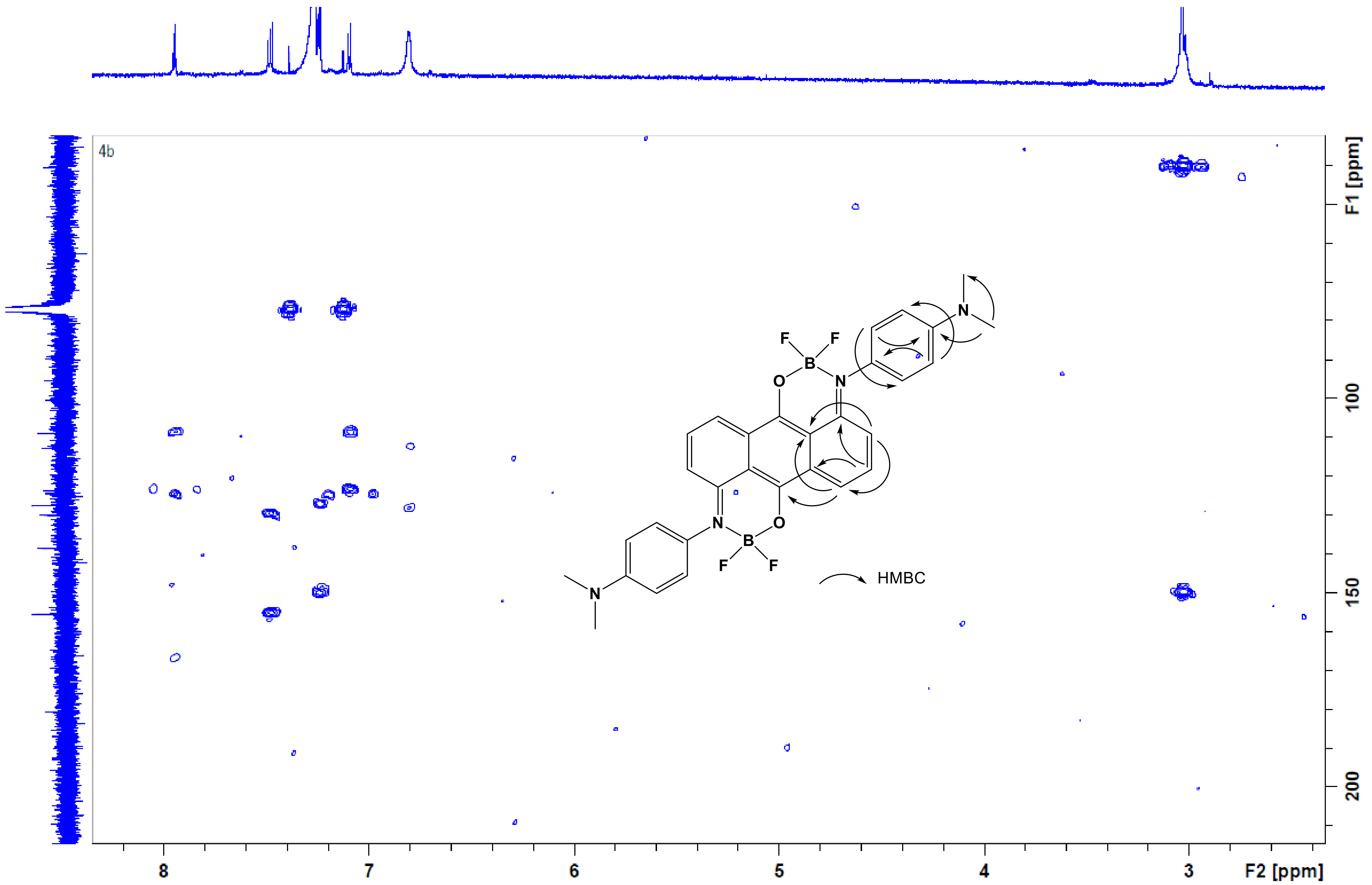

Figure S31. HMBC spectrum of **8** (800 MHz, $CDCl_3$).
